# *AOX1a* Expression in *Arabidopsis thaliana* Affects the State of Chloroplast Photoprotective Systems under Moderately High Light Conditions

**DOI:** 10.3390/plants11223030

**Published:** 2022-11-09

**Authors:** Elena V. Garmash, Olga V. Dymova, Ekaterina V. Silina, Ruslan V. Malyshev, Elena S. Belykh, Mikhail A. Shelyakin, Ilya O. Velegzhaninov

**Affiliations:** Institute of Biology, Komi Science Centre, Ural Branch of Russian Academy of Science, Syktyvkar 167982, Russia

**Keywords:** alternative oxidase, *Arabidopsis thaliana*, *AOX1a*-transformed lines, ascorbate, high light stress, non-photochemical quenching, violaxanthin de-epoxidase

## Abstract

Alternative oxidase (AOX) in the mitochondrial electron transport chain is considered important for sustaining photosynthesis under high light conditions. Here, we examined the effects of the AOX pathway on the state of chloroplast photoprotective systems. *Arabidopsis thaliana* plants (4 weeks old), comprising three genotypes (wild type [WT], overexpressing [XX-2] and antisense [AS-12] lines for *AOX1a*), were exposed to moderately high light conditions (MHL, 400 μmol m^−2^ s^−1^) in a short-term experiment (8 h). After 8 h of MHL, the WT and XX-2 plants showed stable non-photochemical quenching (qN) and violaxanthin cycle activity. Antisense plants displayed the lowest level of qN and a lower de-epoxidation state (DEPS) relative to plants of the same line after 4–6 h MHL, as well as compared to WT and XX-2 plants after 8 h MHL. The decline in DEPS in AS-12 plants was attributed to an insufficient violaxanthin de-epoxidase activity, which in turn was associated with a decrease in reduced ascorbate levels in the chloroplasts and leaves. Simultaneously, gene expression and the activity of ascorbate peroxidase in the antisense line increased after 8 h of MHL, supporting the compensatory effect of the antioxidant system when *AOX1a* expression is suppressed. This study emphasizes the role played by AOX in modulating the photoprotection processes and in the maintenance of relationships between mitochondria and chloroplasts by influencing ascorbate content.

## 1. Introduction

Chloroplasts and mitochondria, the main organelles that provide energy to plant cells, are in contact at the metabolic and signalling levels, and their relationship maintains homeostasis in photosynthetic cells [1,2]. The involvement of components of the mitochondrial electron transport chain (mETC) is considered to optimize photosynthetic processes in plants [3]. In addition to the main cytochrome pathway (CP) for electron transport from ubiquinone to O_2_, the mETC in higher plants contains the cyanide-insensitive alternative pathway (AP) catalysing by the terminal alternative oxidase (AOX) [1]. The AOX is capable of shunting electrons from ubiquinone directly to oxygen, bypassing proton-pumping complexes III and IV; thus, the total efficiency of respiration is reduced. AOX function is important for mitochondrial redox balance maintenance and limiting the formation of ROS [1]. It is also considered that in illuminated green plant tissues, functioning AOX aids in the utilisation of the excessive reducing equivalents transported from the chloroplasts, preventing oxidative damage to photosynthetic organelles and whole cells [1,3,4]. In addition, AOX gene expression is light-induced and mediated through photoreceptor control [5,6].

Experiments in which AOX gene expression was suppressed or stimulated confirmed the importance of AOX activity in supporting efficient photosynthetic performance. Under high light conditions, particularly under additional stress factors (e.g., drought), *aox1a* mutants (*Arabidopsis thaliana* and *Nicotiana tabacum*) or plants treated with CP or AP inhibitors suffered from stress. A decrease in the maximum quantum efficiency of photosystem II (F_v_/F_m_) [7,8], the rate of photosynthetic linear electron flow [9], disturbance in Chl accumulation and plastidial protein import [10], and a more reduced state of ubiquinone (UQ) and plastoquinone (PQ) pools [11] compared with wild type (WT) plants were observed. Conversely, the overexpression of *AOX1a* optimised photosynthesis under high light stress [11,12].

It is shown that *aox1a* mutants can respond to high light, particularly in the presence of CP inhibitors, by increasing non-photochemical quenching of Chl fluorescence (NPQ) [7,9]. One component of NPQ is pH-dependent and involves zeaxanthin (Zx) formation from the de-epoxidation of violaxanthin (Vx) under the action of light [13,14]. Zx is capable of absorbing and dissipating energy from excited Chl. The de-epoxidation of Vx is catalysed by violaxanthin de-epoxidase (VDE), which is activated by low pH in the thylakoid lumen of chloroplasts and uses ascorbate (Asc) as a reductant [13,14]. The last step in Asc synthesis is linked with mETC activity [15]; thus, this association can connect functioning AOX and VDE. A possible relationship between mETC functioning and the activity of xanthophyll pigment conversion has already been noted [16,17,18,19]. In particular, the inhibition of the AOX pathway by salicylhydroxamic acid (SHAM) in Rumex K-1 leaves treated with intense light (600 μmol m^−2^ s^−1^) caused a restriction in photosynthetic linear electron flow and decreased xanthophyll cycle (VXC) de-epoxidation and the induction of NPQ [17].

Among the five AOX genes, *AOX1a* in *A. thaliana* is the most stress-responsive gene based on the highest variety of stress-inducing factors affecting its expression and the magnitude of the expression response, e.g., [7,20,21]. Previously, we found that antisense *A. thaliana* line for *AOX1a* (AS-12) displayed a compensatory upregulation of the majority of AOX-encoding genes and genes of other respiratory process components when exposed to moderately high light (400 μmol m^−2^ s^−1^) [22]. As a result, the ROS concentrations in AS-12 cells were decreased in comparison with the wild type. In the current study, we examined the previously unknown effects of *AOX1a* expression in *A. thaliana* transformed plant lines on the state of chloroplast photoprotective systems. Specifically, the conversion of VXC pigments under increased light conditions was studied to determine the relationship between AOX and VDE activities, and to estimate whether this connection is mediated by Asc level.

## 2. Results

### 2.1. Plants with Altered AOX1a Expression Exhibit Different Respiration Responses to MHL

**Figure 1:**
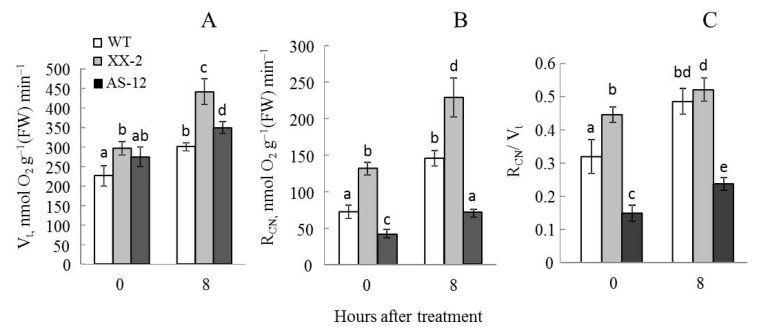
Total respiration rate (V_t_) (**A**), KCN-resistant respiration (R_CN_) (**B**), and relative part of R_CN_ in V_t_ (**C**) in leaves of wild type (WT), *AOX1a*-overexpressing (XX-2), and antisense (AS-12) *Arabidopsis thaliana* plants grown at 90 μmol m^−2^ s^−1^ (0 h) and after 8 h of moderately high light exposure (400 μmol m^−2^ s^−1^). Data are presented as mean ± SE of values from three independent experiments (n = 4–6 for each experiment). Significant differences between mean values (ANOVA, Duncan’s test, *p* < 0.05) are indicated by different letters (a, b, c, d, e). The same or double letters (ab, bd) indicate no significant differences between the means.

The total respiration rate (V_t_) of plants increased in all lines after 8 h of exposure to MHL, but the increase was more noticeable in the XX-2 overexpression line (Figure 1A). The CN-resistant respiration (R_CN_) rates were the highest and the lowest in the leaves of XX-2 plants and AS-12 plants illuminated with 90 µmol m^–2^ s^–1^, respectively, but after MHL treatment R_CN_ increased equally almost two-fold in all three lines (Figure 1B). The relative part of R_CN_ in total respiration after MHL treatment in the WT and XX-2 leaves reached the same values (Figure 1C). Thus, all lines responded to high light with the increase in respiration, and AS-12 plants had the lowest V_t_ and R_CN_ values.

### 2.2. Specific Leaf Weight and Chl Content

SLW and Chl content per dry weight were not significantly different among genotypes and did not change after the short 8 h MHL treatment (Table S1). Chl content per leaf area exhibited a very slight tendency to increase during MHL treatment in all lines, but the values in WT and AS-12 plants decreased to the initial level after 8 h MHL (Table S1).

### 2.3. Effects of MHL on Photosynthetic Apparatus Functioning

**Figure 2:**
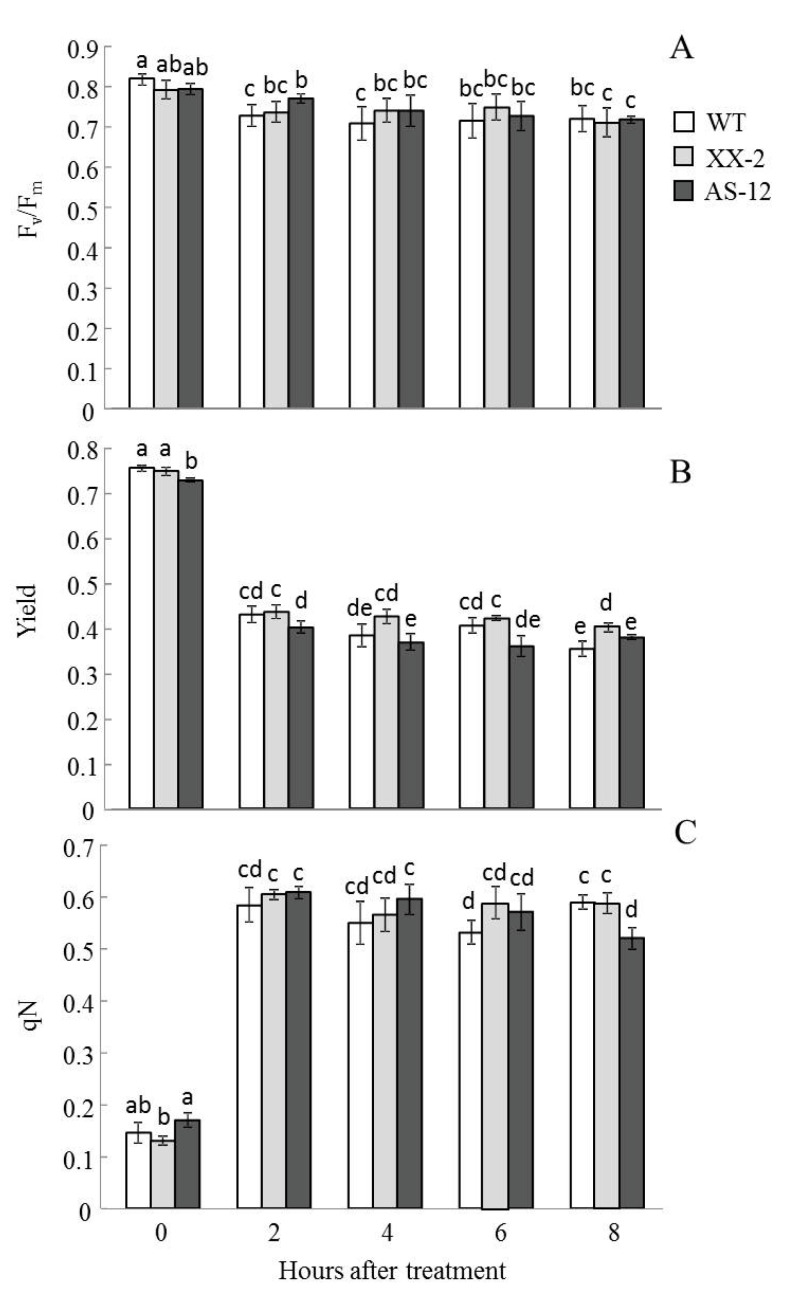
Parameters of Chl fluorescence. Maximum and actual photochemical efficiency of PSII expressed as F_v_/F_m_ (**A**) and Yield (**B**) respectively, and the coefficient of non-photochemical quenching or qN (**C**) in leaves of WT, XX-2, and AS-12 *Arabidopsis thaliana* plants grown at 90 μmol m^−2^ s^−1^ (0 h) and after 2–8 h of moderately high light (400 μmol m^−2^ s^−1^). Data are presented as mean ± SE of values from three independent experiments (n = 6–10 for each experiment). Significant differences between mean values (ANOVA, Duncan’s test, *p* < 0.05) are indicated by different letters (a, b, c, d, e). The same or double letters (ab, bc, cd, de) indicate no significant differences between the means.

No significant differences in the parameters of Chl fluorescence were observed among the three lines when grown at 90 μmol m^−2^ s^−1^ (0 h) (Figure 2). In all the WT samples, the F_v_/F_m_ reached 0.8, which is the typical value for a healthy mature leaf. After 8 h of MHL exposure, the F_v_/F_m_ slightly decreased to approximately 0.73 in all genotypes (Figure 2A). The effective quantum yield of PSII (yield) decreased in all three lines after exposure to MHL, but in XX-2 plants, the yield was slightly higher after 4 h compared with the other two lines (Figure 2B). The qN increased when plants were exposed to MHL. Thereafter qN slightly decreased in AS-12 plants, and after 8 h of MHL was significantly lower than after 2 h of MHL in this line (Figure 2C). WT and XX-2 lines did not differ significantly in qN values. In general, the WT did not differ significantly from AS-12 and XX-2 in their photosynthetic response to MHL except for decreased qN values in AS-12 after 8 h of MHL.

### 2.4. Violaxanthin De-Epoxidation and the Effectiveness of the Violaxanthin Cycle Decreased in AS-12 Plants after 8 h of MHL

**Figure 3:**
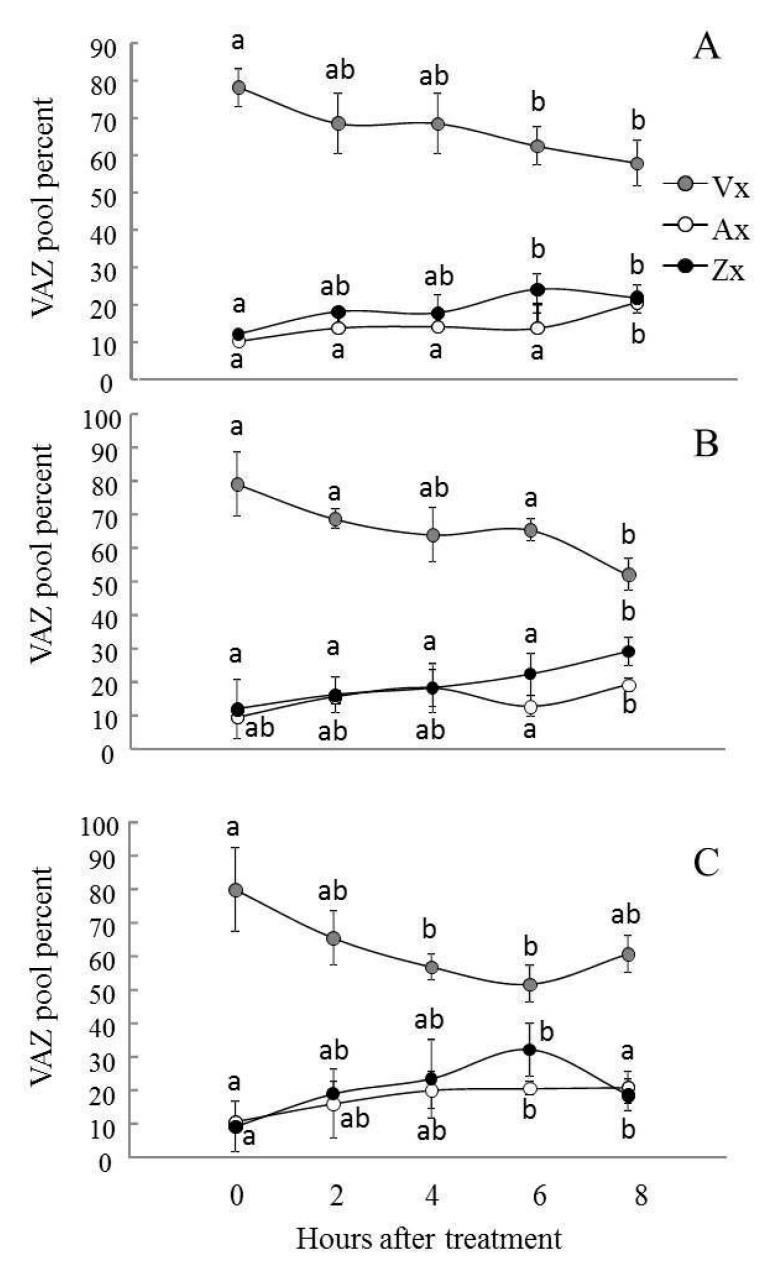
Relative percentage of xanthophyll cycle pigments (Vx, Ax, Zx) in leaves of WT (**A**), XX-2 (**B**), and AS-12 (**C**) *Arabidopsis thaliana* plants grown at 90 μmol m^−2^ s^−1^ (0 h) and after 2–8 h of moderately high light (400 μmol m^−2^ s^−1^). Vx—violaxanthin, Ax—anteraxanthin, Zx—zeaxanthin. Data are presented as mean ± SE of values from three independent experiments (for n = 3 for each experiment). Significant differences between mean values (ANOVA, Duncan’s test, *p* < 0.05) are indicated by different letters (a, b). The same or double letters (ab) indicate no significant differences between the means.

In all three lines exposed to 90 µmol m^–2^ s^–1^ (0 h), the content of absolute and relative Vx was highest in pigments associated with the VXC compared to ones after MHL (Figure 3, Table S2). The Vx content in all three lines constituted 80% of all VXC pigments (VAZ) (Figure 3). The Ax and Zx contents did not exceed 10% of the VAZ pool. As expected, after exposure to MHL, a decrease in absolute Vx content and an increase in Ax and Zx content were observed in all lines. After 4 h of MHL, the relative Vx content was ~60–70% of the VAZ, and the relative content of Zx and Ax increased to 15–20% of VAZ. After 8 h of MHL, the xanthophyll cycle functioning in AS-12 plants differed from that of the other two plant lines. Specifically, the decrease in absolute Vx content and increase in relative Vx content in the VAZ pool coincided with a decrease in absolute Zx concentration and relative Zx levels.

**Figure 4:**
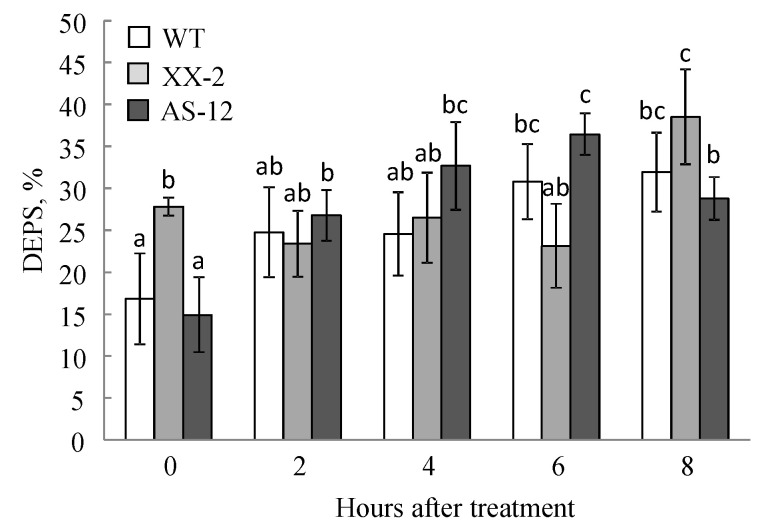
De-epoxidation state (DEPS) of leaves of WT, XX-2, and AS-12 *Arabidopsis thaliana* plants grown at 90 μmol m^−2^ s^−1^ (0 h) and after 2–8 h of moderately high light (400 μmol m^−2^ s^−1^). Data are presented as mean ± SE of values from three independent experiments (n = 3 for each experiment). Significant differences between mean values (ANOVA, Duncan’s test, *p* < 0.05) are indicated by different letters (a, b, c). The same or double letters (ab, bc) indicate no significant differences between the means.

As a result of changes in VXC pigments, the DEPS value changed during the experiment (Figure 4). At the controlled growth light intensity (90 μmol m^−2^ s^−1^) the DEPS value was the highest in the XX-2 line (28%), which was almost two times higher than that measured in WT and AS-12 plants. However, after 2 h of MHL, the DEPS value initially decreased in XX-2 plants and increased after 8 h. In WT and AS-12 plants, the DEPS value increased throughout the MHL exposure, but in AS-12 this parameter decreased after 8 h (Figure 4).

**Figure 5:**
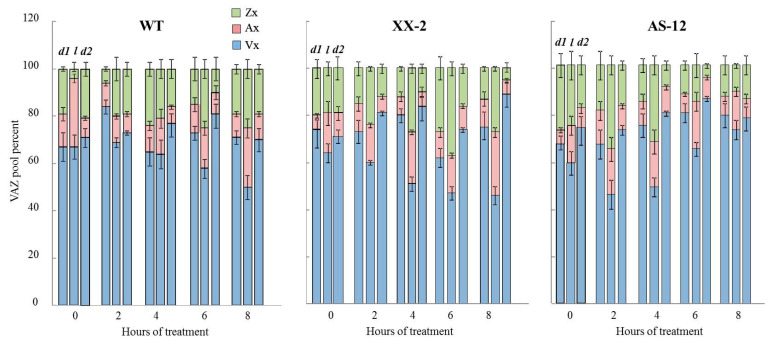
Relative percentage of xanthophylls cycle pigments in leaves of WT, XX-2 and AS-12 *Arabidopsis thaliana* plants grown at 90 μmol m^−2^ s^−1^, i.e., 0 h, and after 2–8 h of moderately high light (MHL; at 400 μmol m^−2^ s^−1^): *d1*—after 1 h of darkness (dark1), *l*—after 1 h of high light exposure (1000 µmol m^–2^ s^–1^) and *d2*—after a subsequent 20 h of darkness (dark2). Time (hours of MHL), experimental conditions (*d1, l, d2*) and genotype were analysed as independent factors. According to factorial ANOVA analysis the effects of the experimental conditions and the genotype are significant at *p* ˂ 0.05 (Wilks’s test) and the time effect is significant at *p* ˂ 0.1 (Wilks’s test).

To examine how the VXC operates in the different genotypes under MHL treatment, we exposed dark-acclimated plants (dark1) to high light (1000 μmol m^−2^ s) and then returned them to darkness (dark2) (Figure 5 and Figure S1). The rate of changes in xanthophyll content during dark-light-dark acclimation reflects the VDE activity in vivo. The absolute values of xanthophylls are presented in Table S3.

Initially, in WT plants grown at 90 µmol m^−2^ s^−1^, Vx made up about 65% of VAZ in dark-acclimated leaves, with ~15% Ax and ~20% Zx (Figure 5). After 1 h of high light illumination, an increase in Ax to 30% of VAZ content was observed, while the Vx content did not change, which indicated that Ax was the preferentially used substrate for the VDE enzyme. After placing plants in the dark for 20 h, the relative content of all pigments returned to their initial levels. In XX-2 and AS-12 plants, under high light the Ax content increased to 15% only, and the Vx content decreased to 60%, indicating a slightly more efficient de-epoxidation in these transformants compared to WT at growth light conditions (90 µmol m^−2^ s^−1^). In all lines, the levels of converted xanthophyll pools gradually increased during the next 6 h of MHL (Figure 5). However, after 8 h of MHL exposure, and following prolonged dark period (dark2), Zx epoxidation of WT plants has slightly decreased; only Ax was used as a substrate for zeaxanthin epoxidase (compared to plants after 6 h of MHL). In the XX-2 after 8 h of MHL, Zx epoxidation was the most effective among lines: the relative content of Vx during high light exposure decreased from ~75 to ~45% and increased to 90% of the VAZ content after the dark2 period. In the AS-12 line after 8 h of MHL, the changes in relative levels of VXC pigments was the lowest during dark-light-dark acclimation, and DEPS did not change and amounted to 20% (Table S3). Thus, AS-12 line after 8 h of MHL reacted by weakening the VXC operation.

### 2.5. Violaxanthin De-Epoxidase Protein Content Follows the NPQ1 Expression

**Figure 6:**
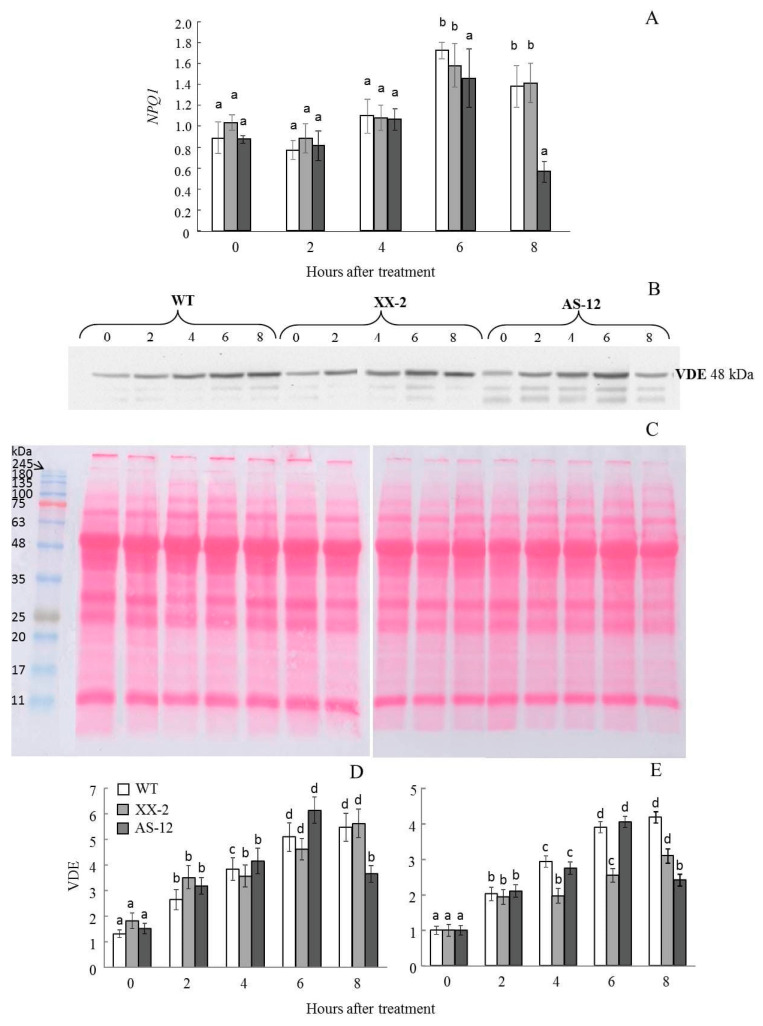
Expression of *NPQ1*, which encodes violaxanthin de-epoxidase (VDE) (**A**), western blot analysis of VDE (**B**), reversible Ponceau S staining control (**C**), and the absolute (**D**) and relative changes (for each line in comparison to those at 0 h) (**E**) in VDE protein levels in leaves of WT, XX-2 and AS-12 *Arabidopsis thaliana* plants grown at 90 μmol m^−2^ s^−1^ (0 h) and after 2–8 h of moderately high light (400 μmol m^−2^ s^−1^). For *NPQ1* expression values, the final number of expression values (ten) was used as n for statistical calculations. The transcript amounts are presented in relation to AT2G28390 and AT4G34270 housekeeping gene [23] expression values. Significant differences between mean values within each gene analysis are indicated by different letters (ANOVA, Duncan’s test, *p* < 0.05). For VDE amount (**C**), data are presented as mean ± SE of values from three replicates. Significant differences between mean values (Kruskal-Wallis test, *p* < 0.05) are indicated by different letters (a, b, c, d). The same letters indicate no significant differences between the means.

The changes in violaxanthin de-epoxidation were attributed to changes in the expression of *NPQ1*, a VDE-coding gene, and VDE protein amount (Figure 6). After plant transfer to MHL, an increased expression of *NPQ1* was detected in all three plant lines (Figure 6A). However, in AS-12 plants, *NPQ1* expression returned to its initial level after 8 h of MHL. VDE protein abundance detected as ~48 kDa band on immunoblots, followed that of *NPQ1* expression (Figure 6B). Plants of all genotypes, before exposure to MHL, contained a low amount of VDE. After exposure to MHL, VDE protein levels increased. After 8 h of MHL, the abundance of VDE in AS-12 plants visibly decreased in immunoblots. Thus, in AS-12 plants after 8 h of MHL, a decrease in the level of gene expression and the amount of VDE protein was observed, which corresponded to a reduced level of VXC functioning.

### 2.6. Changes in the Ascorbate Pool in Leaves and Chloroplasts of A. thaliana WT and Aox1a Lines after MHL

**Figure 7:**
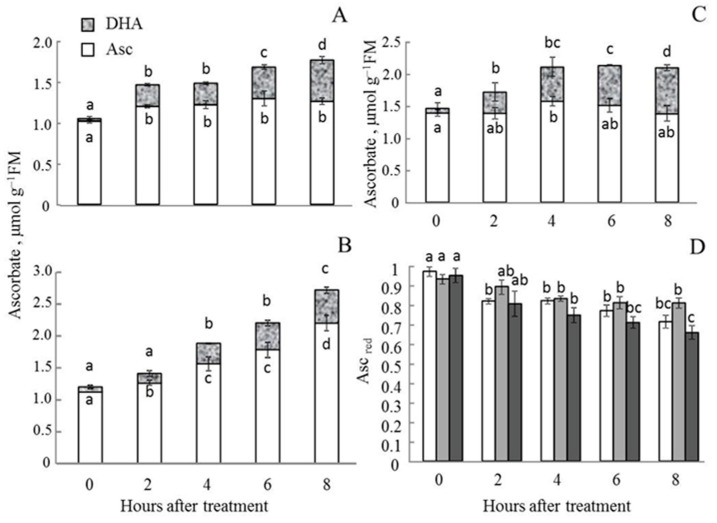
Changes in total cellular reduced and oxidized ascorbate (Asc and DHA, respectively) pools in leaves of WT (**A**), XX-2 (**B**), and AS-12 (**C**) *Arabidopsis thaliana* plants grown at 90 μmol m^−2^ s^−1^ (0 h) and after 2–8 h of moderately high light (400 μmol m^−2^ s^−1^). (**D**) The relative ascorbate content (Asc/(Asc + DHA), Asc_red_). Data are presented as mean ± SE of values from three independent experiments (n = 6–10 from each experiment). Significant differences between mean values (ANOVA, Duncan’s test, *p* < 0.05) are indicated by different letters (a, b, c, d) The same or double letters (ab, bc) indicate no significant differences between the means.

In all lines, the total Asc level gradually increased throughout the experiment (Figure 7). However, in WT and AS-12 plants, absolute and relative concentrations of reduced Asc decreased, while that of the oxidised form of Asc (DHA) significantly increased. The relative Asc content (Asc/(Asc + DHA), Asc_red_) in leaf tissue decreased more significantly in AS-12 than in WT during MHL exposure (Figure 7D). After 8 h of MHL, Asc_red_ in XX-2 was the highest among lines, whereas AS-12 was lower than that in WT.

**Figure 8:**
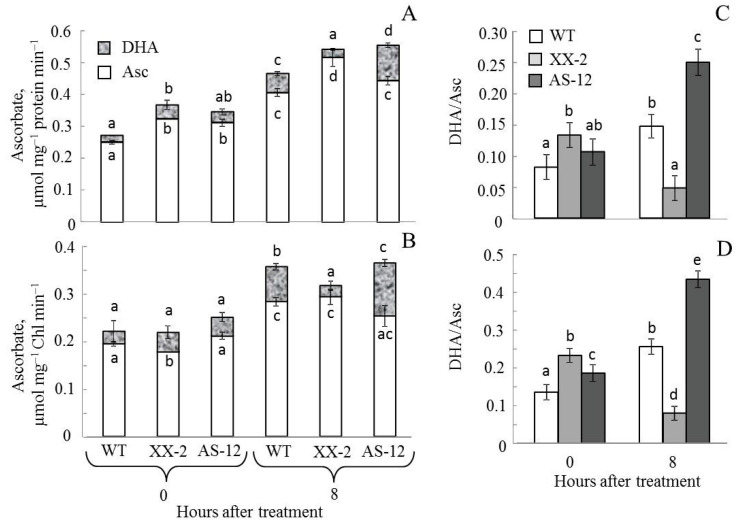
Changes in reduced (Asc) and oxidized (DHA) ascorbate pools per mg of chloroplast protein (**A**) and per mg of Chl (**B**) in chloroplasts isolated from leaves of WT, XX-2, and AS-12 *Arabidopsis thaliana* plants grown at 90 μmol m^−2^ s^−1^ (0 h) and after 8 h of moderately high light exposure (400 μmol m^−2^ s^−1^). (**C**,**D**) The relative ascorbate content (Asc/(Asc + DHA), Asc_red_) per mg of chloroplast protein and per mg of Chl, respectively. Data are presented as mean ± SE of values from three independent experiments (n = 6 for each experiment). Significant differences between mean values (ANOVA, Duncan’s test, *p* < 0.05) are indicated by different letters (a, b, c, d, e). The same or double letters (ab, ac) indicate no significant differences between the means.

In chloroplasts isolated from leaves after 8 h of MHL exposure, the total Asc content per protein and Chl units increased in WT and XX-2 lines compared with that of plants at 0 h (Figure 8) (the content of Chl and protein in isolated chloroplasts is presented in Figure S2). The increase in the total pool of Asc in XX-2 plants was because of Asc accumulation; in the WT and AS-12 plants, the levels of both oxidized and reduced forms of Asc increased (Figure 8A,B). Thus, after 8 h of MHL treatment, the relative levels of Asc (Asc_red_) in XX-2 increased and were the highest among lines; in AS-12, the Asc_red_ value reduced strongly than that in WT and was the lowest among lines (Figure 8C,D). Conversly, the value of DHA/Asc after 8 h of MHL treatment in leaves and chloroplasts, presented in Figure S3, was the lowest in the XX-2 and the highest in the AS-12. In general, the antisense line responded to MHL treatment by reducing the cellular and plastid pool of Asc.

### 2.7. Gene Expression and Activity of Ascorbate Peroxidase in Leaves and Chloroplasts

**Figure 9:**
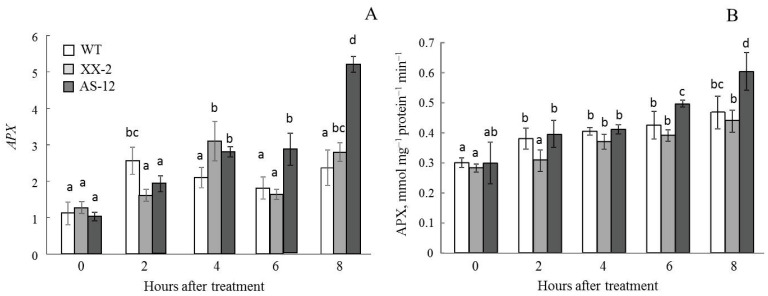
Expression of *APX*, encoding ascorbate peroxidase (**A**), and APX activity (**B**) in leaves of WT, XX-2, and AS-12 *Arabidopsis thaliana* plants grown at 90 μmol m^−2^ s^−1^ (0 h) and after 2–8 h of moderately high light (400 μmol m^−2^ s^−1^). For *APX* expression values, the final number of expression values (10) was used as n for statistical calculations. The transcript amounts are presented in relation to AT2G28390 and AT4G34270 housekeeping gene [23] expression values. Significant differences between mean values within each gene analysis (ANOVA, Duncan’s test, *p* < 0.05) are indicated by different letters (a, b, c, d). The same or double letters (ab, bc) indicate no significant differences between the means. For APX activity, data are presented as mean ± SE of values from three independent experiments (n = 3–6 for each experiment).

In the growth conditions, the *APX* expression level and enzyme activity were low in all plant lines. (Figure 9). After exposure to MHL, these parameters increased. After 8 h of MHL, both the *APX* transcript concentrations and APX activity levels were the highest in AS-12 plants.

**Figure 10:**
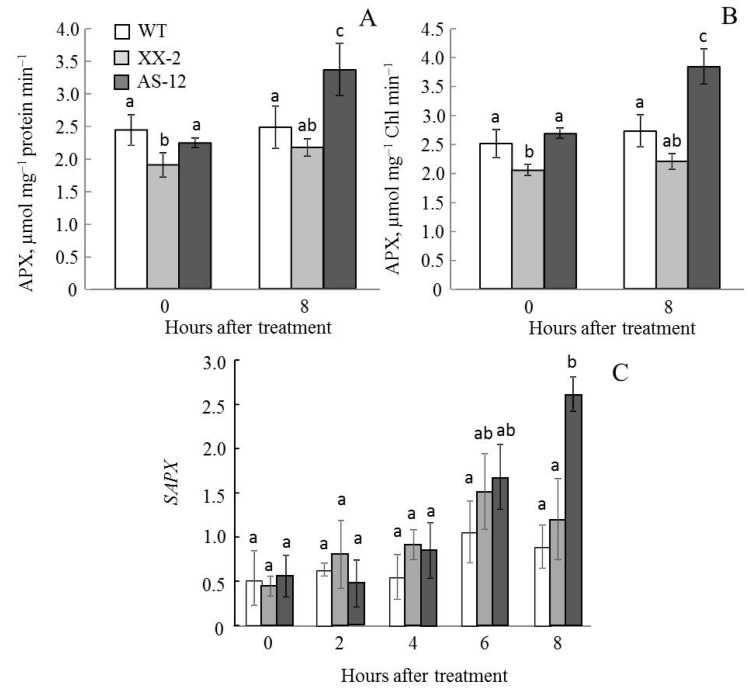
Ascorbate peroxidase (APX) activity in isolated chloroplasts per mg of chloroplast protein (**A**) and per mg of Chl (**B**), and expression of *SAPX*, encoding stromal APX (**C**), in leaves of WT, XX-2, and AS-12 *Arabidopsis thaliana* plants grown at 90 μmol m^−2^ s^−1^ (0 h) and after 8 h of moderately high light (400 μmol m^−2^ s^−1^). For *SAPX* expression values, the final number of expression values (ten) was used as n for statistical calculations. The transcript amounts are presented in relation to AT2G28390 and AT4G34270 housekeeping gene [23] expression values. Significant differences between mean values within each gene analysis are indicated by different letters (ANOVA, Duncan’s test, *p* < 0.05). For APX activity, data are presented as mean ± SE of values from three independent experiments (n = 3–6 for each experiment). Significant differences between mean values (ANOVA, Duncan’s test, *p* < 0.05) are indicated by different letters (a, b, c). The same or double letters (ab) indicate no significant differences between the means.

In the same way, the APX activity in chloroplasts and the expression of *SAPX*, a gene encoding a stromal APX form, in AS-12 plants after 8 h of MHL significantly increased and were the highest among the lines (Figure 10).

Thus, the antisense line responded to MHL treatment with an increased gene expression and activity of APX both in leaves and chloroplasts.

## 3. Discussion

*Arabidopsis thaliana* plants were exposed to MHL (400 µmol m^–2^ s^–1^). This illumination is in the upper part of the light-response curve, i.e., the transition from the light-limited increasing phase to the light-saturated plateau for net photosynthesis of the mature rosette leaf [22]. Thus, the light conditions were on the edge of the transition from physiologically acceptable to stressful. This light condition was chosen to determine the specific mechanisms by which the metabolic balance in plants is maintained with altered alternative oxidase.

Plant lines differed in their respiratory activity (Figure 1), which was obviously due to the differences in *AOX1a* expression levels [24]. Among lines, XX-2 plants showed the maximal values of V_t_ and R_CN_, AS-12—minimal ones. All plant lines reacted to MHL treatment by higher rates of the total and the CN-resistant respiration. However, the increasing in R_CN_ in lines was corresponded to a higher expression of different AOX genes showed in our previous study [22]. The higher respiration activity of XX-2 plants was correlated with the high, stable levels of *AOX1a* expression, WT plants reached the same relative part of R_CN_ as the XX-2 line, but showed increased expression of three AOX genes (*AOX1a*, *AOX1c*, and *AOX2*) [22]. The AS-12 line reacted by increasing the expression of another AOX gene, *AOX1d* [22]; respiration also increased as a result of both CN-resistant and CN-sensitive respiratory components, as also observed in WT and XX-2 lines.

It is considered that AOX1D, which is highly responsive to stress, could not provide full compensation for the absence of AOX1A owing to different mechanisms of activation of the protein [25,26]. It is confirmed by the fact that AS-12 plants displayed a strong compensatory effect, typically after 8 h of MLT, by up-regulating the expression of the majority of genes encoding other respiratory components [22]. It was also shown that in the AS-12 plants, the AP works at its full capacity even in the absence of stress; therefore, there was no capability for increasing AP activity in vivo even after high light treatment [27]. The importance of AOX for mitochondrial flexibility is more noticeable in double mutants of cyt *c* and AP components; these plants experience more stress than plants with the respective single mutants under adverse conditions [28].

Increased light intensity affected chloroplast defence mechanisms similarly in all three plant lines; the actual photochemical efficiency of PSII (yield) decreased and qN of Chl fluorescence increased. However, the Yield was slightly higher in XX-2 plants than in other lines. In earlier studies of the XX-2 line, a stable level of photosynthesis, a higher coefficient of photochemical quenching (qP), and no increase in photoinhibition were observed after 8 h of high light treatment (800 µmol m^−2^ s^−1^) [27]. The ability of different *AOX1a*-overexpressing plant species to withstand high light stress has been revealed in other studies [11,12,22].

The AS-12 plants did not show any visible photoinhibition symptoms; however, qN values after 8 h of MHL were slightly lower than that of XX-2 and WT plants. In another study, the AS-12 plants and *aox1a* plants exposed to a higher light intensity (800 and 1000 µmol m^−2^ s^−1^, respectively) for 8 h showed a significant increase in photoinhibition [5,27], primarily because of increased chronic photoinhibition [27]. It had been previously observed that PSI and PSII functioning in the leaves of *A. thaliana aox1a* plants decreased under high light (600 µmol m^−2^ s^−1^) exposition [9]. Additionally, the UQ and PQ pools in *aox1a* plants after exposure to 650 µmol m^−2^ s^−1^ light were increased to similar levels [29]. This, in turn, favours the formation of potentially damaging ROS and photoinhibition. The redox state of chloroplasts affects *АОХ * expression and other mitochondrial dysfunction stimulon genes, but it is supposed that mitochondrial dysfunction accompanied by a disturbance of electron transport to oxygen is a primary condition for induction of gene expression. The integration of signals is one of the instruments of coordination of metabolic interactions between organelles. Most likely, a key signal of this integration is ROS generated by both organelles [1,2].

Changes in qN are caused by the activation of excess light energy dissipation systems in chloroplasts. Among these, the VXC plays a key role [13,14]. Interestingly, in AS-12 plants after 8 h of MHL, the relative content of Vx (within the total VAZ value) increased from 50% to 60%, and the relative content of Zx decreased from 30% to 20%, but the Ax content did not change, indicating an incomplete transition (de-epoxidation) (Figure 3). As a result, the DEPS value decreased (Figure 4). *NPQ1* expression, VDE abundance, and VDE activity in vivo (the latter deduced from the xanthophyll cycle operating during dark-light-dark acclimation) also decreased in the AS-12 plants after 8 h of MHL. Therefore, it can be assumed that the decrease in de-epoxidation level was due to insufficient VDE activity. Since SLW and the Chl content per dry weight and leaf area were not significantly different among genotypes and after short 8 h MHL treatment (Table S1), the observed changes in xanthophyll cycle operation are assumed to be related to the level of *AOX1a* expression. It is also confirmed by two-way ANOVA analysis according to which the exposure of all lines for 8 h of MHL caused changes in DEPS, which were due to differences in the level of *AOX1a* expression (Table S4).

The decrease in VDE activity could be related to Asc availability; VDE requires Asc as a reductant [14]. Indeed, after 8 h of MHL, the relative content of the reduced Asc pool decreased and the ratio DHA/Asc increased in the leaves and isolated chloroplasts of AS-12 plants (Figure 7 and Figure 8). The final stage of the ‘Smirnoff–Wheeler’ (L-galactose) pathway, the key Asc biosynthesis system, is linked with mETC. L-galactono-1,4-lactone dehydrogenase (GLDH), the enzyme that converts galactone (GL) to Asc, and also delivers electrons to cyt *c* [15]. Additionally, the CP and AP pathways of electron transport in mitochondria both play important role in Asc synthesis. In particular, the increased capacity of the AOX metabolic system favours the synthesis of Asc by promoting a more oxidised state of the cyt *c* pool, particularly in high light conditions. That shift is thought to prevent the over-reduction of mitochondrial transporters under such conditions [15]. Additionally, there is a feedback AOX activity and metabolites of the ascorbate synthesis. The capacity of alternative respiration was strongly decreased by GL probably mediated by redox-inactivation of AOX enzyme [30]. Alternative respiration was shown to be the key factor that helps support Asc synthesis in dysfunctional mitochondria. Thus, a reduced amount of AOX protein in AS-12 plants could adversely affect Asc synthesis.

There are important findings that AOX isoforms can be dual localized in the chloroplast stroma and the mitochondrion [31]. AOX1b and AOX2 were revealed to rescue the variegation phenotype of the Arabidopsis PTOX deficient mutant (*im*). While partly dual targeting of AOX1b and AOX2 is possible, this supposed not to reduce the adverse effects of *AOX1a* absence: *AOX1d*, but not other AOX family genes expression increased in AS-12 plants as a result of MHL exposure [22]. This may be the subject of future research.

In experiments using Arabidopsis *vtc2* (*VTC2* encodes GDP-L-galactose phosphorylase/guanyltransferase, one of the important L-galactose pathway enzymes that participates in the synthesis of Asc) and *npq1* mutants, the ROS-scavenging capability of Asc was shown to be more important than Asc participating in the VXC and NPQ during acclimatisation to high light conditions [32]. It is hypothesised that the Asc levels might determine the de-epoxidation capacity when conditions arise that restrict Asc synthesis, such as in our experiments when the functionally competent protein AOX was absent.

Ascorbate (along with glutathione) is the main metabolite of the ascorbate-glutathione cycle (AGC), serving as an electron donor for APX, which catalyses the reduction of H_2_O_2_ to water [33]. The AGC operates in all cellular compartments. In chloroplasts, this cycle removes large amounts of H_2_O_2_ generated during photosynthesis. Additionally, dual targeting of many proteins, including enzymes of the AGC, occurs in the plant cell [34], which can expand the function(s) of a protein. More specifically, a protein located in more than one location will presumably function with a distinct biochemical process in each location [35]. In green leaves, the mitochondrial AGC has been proposed to function in coping with photosynthetic and environmental stress-induced oxidative stress [36]. Therefore, we studied the expression of genes encoding both cytosolic and stromal forms of APX and the activity of the enzyme in leaf tissue and chloroplasts. All these parameters were higher in AS-12 plants after 8 h of MHL compared to that of WT and XX-2 (Figure 9 and Figure 10). Thus, APX effectively operated in the cell and chloroplasts, and this APX functioning was determined by the level of *AOX1a* expression. This could also indicate the activation of compensatory mechanisms of antioxidant defence in the AS-12 plants. It has been reported, including in our studies, that the stable induction of non-phosphorylating respiratory pathway component genes and genes encoding enzymes with antioxidant capability occurs in response to AOX gene suppression [21,22,37].

## 4. Conclusions

**Figure 11:**
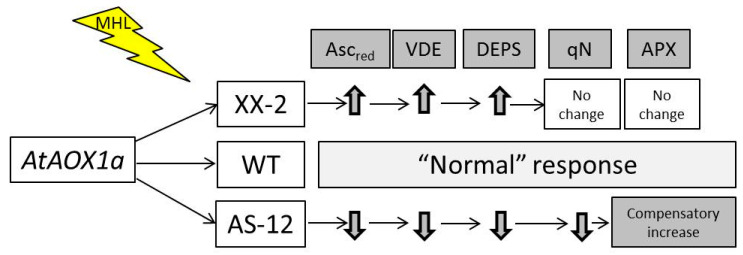
Physiological response of *AOX1a*-overexpression (XX-2) and *AOX1a*-antisense (AS-12) *Arabidopsis thaliana* plants versus the “normal” wild type (WT) response after 8 h of exposure to moderately high light (MHL). Asc_red_—relative levels of reduced ascorbate (from total pool of ascorbate), VDE—violaxanthin de-epoxidase content and gene expression, DEPS—level of de-epoxidation, qN—non-photochemical quenching, and APX—ascorbate peroxidase activity. Down and up bold arrows indicate a decrease and increase in a parameter, respectively. Arrows to the right indicate the possible sequence of events in the cell at different levels of *AOX1a* expression. See the text for further explanation.

The data obtained here suggest that changes in the level of *AOX1a* expression determine the degree of involvement of the photosynthetic defence system. Even under moderate stress conditions, AOX plays an important role in maintaining the metabolism of photosynthesising cells. The plant response and possible sequence of events in cells with different *AOX1a* expression levels are presented in Figure 11. Normal and overexpressed levels of *AOX1a* provide stability to photosynthetic and photoprotective processes. However, WT plants exhibited the accumulation of cellular and chloroplast-oxidized Asc, which can be regarded as a symptom of oxidative stress development. The suppression of *AOX1a* in antisense plants increases cellular and chloroplast antioxidant defence. The decrease in the level of de-epoxidation was most likely caused by an insufficient VDE activity owing to a reduction in the availability of Asc (namely, the relative content of Asc, Asc_red_), which is a strong antioxidant. In a short-term experiment, we found that changes in the expression and activity of mitochondrial AOX affect the functioning of the photoprotective and antioxidant systems in chloroplasts, and signals of mitochondrial dysfunction reach the chloroplasts relatively quickly. Future studies should clarify the relationship between AOX and the xanthophyll cycle, which function as key protective energy-dissipating systems in the mitochondria and chloroplasts of photosynthesising cells, respectively.

## 5. Materials and Methods

### 5.1. Plant Material and Growth Conditions

Three lines of *Arabidopsis thaliana* were used: wild-type (WT) Columbia-0, *AtAOX1a* antisense AS-12, and *AtAOX1a* overexpression XX-2 (for additional information, see [24]). Seeds of *A. thaliana* were obtained from the Nottingham Arabidopsis Stock Centre (NASC, Nottingham University, Nottingham, UK); stock numbers were N6707 for AS-12 and N6591 for XX-2 plant seeds. Seeds of all three lines were planted in pots filled with pre-wetted perlite:vermiculite:soil (1:1:2) mix. After sowing, the seeds were stratified at 4 °C in the dark for 96 h before transferring the pots to a growth chamber, where they were maintained under a controlled temperature of 22 °C. The day length was 10 h (from 7:00 to 17:00), and the photosynthetic active photon flux density was 90 μmol photons m^−2^ s^−1^. The light was provided by luminescent lamps (TL-D 30 W and TL-D 30 W Aquarelle, Philips, Amsterdam, The Netherlands).

After 4 weeks (growth stage 1.14; [38]), the plants were exposed to moderately high light (MHL; 400 μmol m^−2^ s^−1^) for 8 h. Plants were transferred at the same time to the MHL condition 2 h after the beginning of the growing photoperiod (at 9.00 a.m.)

The leaves were collected from a pool of plants, and the fully developed leaves were retained for subsequent analyses. Leaf samples for respiration measurement and chloroplast isolation were obtained after 0 and 8 h of exposure to the higher irradiance. For all other measurements, leaf samples were collected after 0, 2, 4, 6 and 8 h of MHL exposure. Leaves collected at 0 h were considered the control samples (normal light; 90 μmol m^−2^ s^−1^).

### 5.2. Specific Leaf Weight

Leaf samples (20 in total) were weighed and dried to the air-dry state at 70 °C. The leaf area was measured using prints on paper. The specific leaf weight (SLW) was calculated by dividing the dry weight of leaves by their surface area (g DW dm^−2^).

### 5.3. O_2_ Exchange and CN-Resistant Respiration in Leaf Tissues

For measurement of the O_2_ uptake rate, a Clark-type thermoelectrically controlled oxygen electrode (Oxytherm System, Hansatech Inst., Pentney, Norfolk, UK) was used. All measurements were conducted at 21 °C. Leaf tissue samples (0.02 g wet weight) were transferred into the reaction vessels of the electrode filled with 1.5 mL HEPES buffer (50 mM, pH 7.2), and the total respiration (V_t_) was measured. To measure CN-resistant respiration (RCN), similar measurements were conducted in the presence of the cytochrome respiration inhibitor KCN (2 mM).

### 5.4. Gene Expression Analysis

Gene expression was analysed using real-time quantitative reverse-transcription PCR (qRT-PCR) and a CFX96 thermal cycler (Bio-Rad, Hercules, CA, USA). Rosette leaves were collected from plants, flash-frozen in liquid nitrogen, and then stored at −80 °C before analysis. Total RNA was isolated using an Aurum Total RNA Mini Kit (Bio-Rad). Following the manufacturer’s instructions, polyvinylpyrrolidone (2%, *w*/*v*) was added to the lysis solution. RNA concentrations were determined using a Qubit™ RNA HS Assay Kit (Thermo Fisher Scientific, Waltham, MA, USA). Samples of total RNA (1 μg) were reverse-transcribed using oligo-dT primers and an MMLV RT Kit (Evrogen, Russia). qRT-PCR assays were performed using qPCRmix-HS SYBR (a PCR ready-mix; Evrogen). Gel electrophoresis was performed to confirm the absence of non-specific PCR-products. Primer-BLAST online tool [39] was used to design primer pairs for each of the genes studied such that they would simultaneously amplify all known splice variants (Table 1); primer efficiency varied from 1.97 to 2.1. Relative expression values were obtained using the 2^−ΔΔC^_T_ method [40]. The target gene expression was normalized to that of two housekeeping genes, AT2G28390 and AT4G34270 [23]. Each experiment was repeated five times with cDNA samples prepared independently for each replication. Each sample contained RNA isolated from 3–5 individual plants. In each experiment, the assay was repeated two times, giving a total of 10 replications per experimental variant.

### 5.5. SDS-PAGE and VDE Western Blot Analysis

For protein gel blot assays, protein extracts from leaves were used. After mixing the protein extracts 1:4 (*v*/*v*) with a modified Laemmli’s sample buffer (0.5 M Tris–HCl [pH 6.8], 20% glycerol [*v*/*v*], 10% SDS [*w*/*v*], 1 mM EDTA, 0.05% bromophenol blue [*w*/*v*]), the samples were boiled for 5 min.

The solubilized proteins (15 mg protein/lane) were electrophoretically separated in a 12.5% polyacrylamide (*v*/*v*) gel using a Mini-PROTEAN III Cell (Bio-Rad) following the standard protocol. Protein content was measured following the Bradford method, using BSA as a standard [41]. Following separation, the proteins were electroblotted onto nitrocellulose membranes in transfer medium (25 mM Tris, 192 mM glycine, 10% methanol [*v*/*v*] and pH 8.3) using a Mini Trans-Blot^®^ Electrophoretic Transfer Cell (Bio-Rad). The cell settings for transfer were 100 V for 90 min at 4 °C. Rabbit polyclonal antisera against VDE (AS15 3091, 1:2000, Agrisera) were used. For evaluating the content of VDE, Ponceau S reverse staining was performed to check for equal loading of the gels. Horseradish peroxidase-linked anti-rabbit secondary antibodies were used for chemiluminescence signal detection, which was quantified using a ChemiDoc XRS Gel Imaging System (Bio-Rad) and Quantity One^®^1-D Analysis software (v.4.6.9, Bio-Rad).

### 5.6. Chl Extraction and Quantification

Leaf samples (about 0.1–0.15 g) were collected and frozen in liquid N_2_. Chl was extracted in 100% chilled acetone and the concentration was estimated spectrophotometrically using a UV-1700 spectrophotometer (Shimadzu, Tokyo, Japan) by measuring the absorbance at 662 nm (Chl a) and 644 nm (Chl b), according to [42].

### 5.7. Carotenoid Analysis and Xanthophyll Cycle Activity Measurement

Fresh leaf samples (approximately 0.1–0.15 g) were flash-frozen in liquid nitrogen and stored at −80 °C before analysis. Quantification of β-carotene (β-car) and xanthophylls (neoxanthin, Nx; violaxanthin, Vx; antheraxanthin, Ax; lutein, Lx; and zeaxanthin, Zx) was performed using reversed-phase high-performance liquid chromatography (HPLC; Knauer, Germany) with an end-capped Diasphere-110-C18NT 5 μm column (250 × 4.0 mm). For HPLC separation, a gradient procedure using eluent A (acetonitrile:methanol:water, 75:12:4, *v*/*v*) and eluent B (methanol:ethyl acetate, 68:32, *v*/*v*) and a flow rate of 2 mL min^−1^ with a total elution time of 34 min was performed. Carotenoid compound identification was based on individual pigment absorption spectra and their individual retention times. Quantitative data on each carotenoid concentration was obtained by comparison of area under the curve (AUC) measurements in sample chromatograms following absorption readings at 440 nm and AUC of chromatograms of the xanthophyll standards at the same wavelength.

Concentrations of carotenoids were expressed as mg g^−1^ leaf DW. For calculation of the de-epoxidation state (DEPS) values for xanthophyll cycle compounds (Vx, Ax, and Zx), the method of [43] was used, where DEPS = (Zx + 0.5 Ax)/(Zx + Ax + Vx). A coefficient ≥ 0.5 indicated that the Ax contained an epoxy group in one of two β-ionone rings. All analyses were performed in triplicate on different samples, with two chromatographic assays for each sample.

For VXC activity, plants were kept in darkness for 1 h, then exposed to white light (1200 µmol m^–2^ s^–1^) for 1 h, and returned to darkness for 20 h (for full Zx epoxidation). Sets of leaves were collected for HPLC analysis before and after the light exposure as well as after the subsequent darkness.

### 5.8. Chl Fluorescence Measurements

A portable pulse-amplitude modulation (PAM) fluorometer (PAM-2100, Walz, Germany) was used to measure Chl fluorescence, following the manufacturer’s instructions. For each light treatment, 8–10 measurements were carried out using leaves from different individual plants. Measurements were conducted at 90 and 400 µmol m^–2^ s^–1^ light intensity for control plants and plants after exposure to high irradiance, respectively. At the beginning of the measurements, leaf samples were incubated in the dark for 30 min and then a saturating light pulse at 8000 µmol m^–2^ s^–1^ was applied for 1 s to measure the maximum quantum yield of PSII (F_v_/F_m_). The F_o_ values were measured using a weak red light modulated at 600 Hz. Maximal fluorescence (*F*_m_) was measured after a short induction pulse (0.8 s) of saturating light at 8000 µmol m^–2^ s^–1^. Then, stationary (F_t_), minimal (F_o_’) and maximal (F_m_’) Chl fluorescence in leaves adapted to the actinic light (90 or 400 µmol m^–2^ s^–1^) were determined (F_m_’ was obtained with a light-saturating pulse at 8000 µmol m^–2^ s^–1^). The F_v_/F_m_ value was calculated as (F_m_ − F_o_)/F_m_. The actual photochemical efficiency of PSII (yield) was determined as (F_m_’ − F_t_)/F_m_’. After depressing the yield key on the PAM-2100 Fluorometer, the system automatically turns on the measuring light (90 or 400 µmol m^–2^ s^–1^), measures F_t_, and immediately afterwards applies a saturation pulse to assess F_m_’. Non-photochemical quenching (qN) of Chl fluorescence was calculated as (F_m_ − F_m_’)/(F_m_ − F_o_).

### 5.9. Measurement of Ascorbate and Dehydroascorbate Levels

The extraction of Asc was conducted with fresh leaf samples (0.5 g fresh weight [FW]) and Asc activity assayed according to [44]. This assay is based on Asc reducing Fe^3+^ to Fe^2+^ and the formation of Fe^2+^ with 2.2′-dipyridyl complexes, which can be quantified spectrophotometrically. To convert dehydroascorbate (DHA) to Asc, the samples were pre-incubated with dithiothreitol (DTT); excess DTT was removed using N-ethylmaleimide (NEM, Sigma-Aldrich, Burlington, MA, USA), and the total Asc concentration was assayed as previously described. The concentration of DHA was determined by calculating the difference between the total Asc levels assayed and the Asc levels in samples without DTT pretreatment. The absorbance of the solution was measured at 525 nm and the results are presented in μmol g^−1^ FW units.

### 5.10. Analysis of Ascorbate Peroxidase Activity

Ascorbate peroxidase (APX) activity was measured in the supernatant of leaf samples (0.5 g FW) after homogenising on ice (2–4 °C) in 0.05 M sodium phosphate buffer (pH 7.0) and centrifuging at 15,000× *g* at 4 °C for 20 min. The assay was according to [45] and based on Asc oxidation over time measured at 290 nm; the results were calculated using the extinction coefficient of 2.8 mM^−1^ cm^−1^ and expressed as mmol of oxidised Asc per milligram of protein per minute (mmol mg^−1^ min^−1^).

### 5.11. Chloroplast Isolation

Intact chloroplasts were isolated using the method described by [46] with small modifications. Leaves (2–3 g FW) were homogenised in precooled chloroplast isolation buffer containing 0.33 M sorbitol, 50 mM HEPES (pH 7.5), 5 mM EDTA, 5 mM EGTA, 1 mM MgCl_2_, 10 mM NaHCO_3_, and 0.5 mM DTT using a pestle and mortar. The homogenate was filtered through four layers of nylon mesh into centrifuge tubes. After centrifugation at 1000× *g* for 10 min, the supernatant was discarded and the pellet was gently resuspended in precooled resuspension buffer containing 0.33 M sorbitol, 0.2 M HEPES (pH 7.0), 4 mM MgCl_2_, and 5 mM NaCl, and stored on ice until required. All steps in the isolation of intact chloroplasts were performed at 4 °C.

### 5.12. Determination of Chl and Protein in Isolated Chloroplasts

Chl was extracted from the chloroplast suspension using 100% chilled acetone and estimated spectrophotometrically [42]. Chloroplast protein content was determined by the Bradford method of [41] using BSA as a standard.

### 5.13. Determination of Ascorbate and Dehydroascorbate Content in Isolated Chloroplasts

Samples of freshly prepared chloroplast suspensions containing 30 μg of Chl were mixed with 10% (*w*/*v*) trichloroacetic acid. Samples were placed on ice for 5 min; then the soluble fraction was neutralised with 0.5 M NaOH to determine Asc and DHA levels according to [47]. For Asc analysis only, 150 mM sodium phosphate buffer (pH 7.4) and water were added to a portion of the neutralised extract. The second portion of the neutralised extract was used to measure total Asc (Asc + DHA) by incubating with 10 mM DTT, 150 mM sodium phosphate buffer (pH 7.4), and 0.5% (*w*/*v*) N-ethylmaleimide for 15 min at room temperature 22–23 °C. Then both samples were vortexed and incubated at room temperature for 1 min. To each was added 10% (*w*/*v*) trichloroacetic acid, 44% (*v*/*v*) H_3_PO_4_, 4% (*w*/*v*) bipyridyl in 70% (*v*/*v*) ethanol, and 3% (*w*/*v*) FeCl_3_. After vortexing, the samples were incubated at 37 °C for 60 min before recording their optical density at 525 nm. The concentration of DHA was calculated by subtracting the concentration of Asc from the total content of the ascorbate pool. A standard curve was constructed for Asc in the range of 0–60 µM.

### 5.14. Measurement of Ascorbate Peroxidase Activity in Isolated Chloroplasts

APX activity was determined as the decrease in absorbance at 290 nm due to Asc oxidation [48]. The reaction mixture contained 50 mM potassium phosphate buffer (pH 7.0), 0.2 mM Asc, and 0.1 mM hydrogen peroxide. The reaction was initiated by the addition of chloroplast suspension (20 µg protein). To avoid loss of chloroplast APX activity in the absence of Asc, isolated intact chloroplasts were placed in a reaction mixture containing 0.5 mM Asc.

### 5.15. Statistical Analysis

The results are presented as mean values with standard errors (SE) (n = 3–15) from at least three independent experiments. After checking for normal distribution of variables, data were analysed using ANOVA followed by parametric Duncan’s test (*p* < 0.05) and Wilks’s tests (*p* < 0.05) or nonparametric Kruskal–Wallis test (*p* < 0.05). The statistical analysis was conducted using the Statistica software (v.6.1, StatSoft. Inc., Tulsa, OK, USA).

## Figures and Tables

**Table 1:** A list of primer sets for qRT-PCR analysis.

Gene Name	AGI No.	Gene Description **	Forward Primer (5′~3′) Reverse Primer (5′~3′)	Product Length, bp
*NPQ1*	At1g08550	Non-photochemical quenching 1	CGAGTGTGCTGTGTCGAGAA TGGCAGTCGAAGGCATCAAA	168
*APX1*	At1g07890	Ascorbate peroxidase 1, cytosolic	TGGCCCTGACATTCCTTTCC ATGTGGGCCTCAGCGTAATC	393
*SAPX*	At4g08390	Stromal ascorbate peroxidase	GCGTCGGTGAATCGGAGTTT CCACCTCTTTGTGGCCATTC	135
*AT2G28390* *	At2g28390	SAND family protein	AACTCTATGCAGCATTTGATCCACT TGATTGCATATCTTTATCGCCATC	61
*AT4G34270* *	At4g34270	TIP41-like family protein	GTGAAAACTGTTGGAGAGAAGCAA TCAACTGGATACCCTTTCGCA	61

Note: *—Housekeeping genes; primers designed and validated in [23]. **—Source—NCBI.

## Data Availability

Not applicable.

## Supplementary Materials

The following supporting information can be downloaded at: https://www.mdpi.com/article/10.3390/plants11223030/s1, Figure S1: Relative percentage of xanthophylls cycle pigments (Vx, Ax, Zx) in leaves of WT, XX-2, and AS-12 *Arabidopsis thaliana* plants grown at 90 μmol m^−2^ s^−1^, i.e., 0 h, and after 2–8 h of moderately high light (MHL; at 400 μmol m^−2^ s^−1^): *d1*—after 1 h of darkness (dark1), *l*—after 1 h of high light exposure (1000 µmol m^–2^ s^–1^) and *d2*—after a subsequent 20 h of darkness (dark2). Vx—violaxanthin, Ax—anteraxanthin, Zx—zeaxanthin. Data are presented as mean ± SE of values from three independent experiments (n = 3 for each experiment). Significant differences between mean values (Kruskal-Wallis’s test, *p* ˂ 0.05) are indicated by different letters (a, b). The same or double letters (ab) indicate no significant differences between the means. Figure S2: Chlorophylls (a + b) (Chl) (A) and protein content (B) in suspension of chloroplasts isolated from leaves of WT, AS-12, and XX-2 *Arabidopsis thaliana* plants grown at 90 μmol m^−2^ s^−1^ (0 h) and after 8 h of moderately high light (at 400 μmol m^−2^ s^−1^). Data are presented as mean ± SE of values from three independent experiments (n = 4–6 for each experiment). Significant differences between mean values (ANOVA, Duncan’s test, *p* < 0.05) are indicated by different letters (a, b). The same or double letters (ab) indicate no significant differences between the means.; Figure S3. The ratio of DHA/Asc in leaves tissue (A) and in suspension of chloroplasts (B,C) isolated from leaves of WT, AS-12, and XX-2 *Arabidopsis thaliana* plants grown at 90 μmol m^−2^ s^−1^ (0 h) and after 8 h of moderately high light (at 400 μmol m^−2^ s^−1^). B and C—DHA/Asc per mg of chloroplast protein and mg of Chl, respectively. Data are presented as mean ± SE of values from three independent experiments (n = 4–6 for each experiment). Significant differences between mean values (ANOVA, Duncan’s test, *p* < 0.05) are indicated by different letters (a, b, c, d). The same or double letters (ab, cd) indicate no significant differences between the means. Table S1: Specific leaf weight and chlorophylls (a + b) (Chl) content on dry weight (DW) and area in leaves of WT, AS-12, and XX-2 *Arabidopsis thaliana* plants grown at 90 μmol m^−2^ s^−1^ (0 h) and after 2–8 h of moderately high light, MHL (at 400 μmol m^−2^ s^−1^). Data are presented as mean values ± SE of values from three independent experiments (n = 20 for SLW, n = 5 for Chl from each experiment). Significant differences between mean values (if there are any) are indicated by different letters (ANOVA, Dunkan test, *p* < 0.05); Table S2: Amounts of pigments (mg g^-1^DW) of the xanthophylls cycle in leaves of wild type (WT), XX-2, and AS-12 *Arabidopsis thaliana* plants grown at 90 μmol m^-2^ s^-1^ (0 h) and after 2-8 h of moderately high light (at 400 μmol m^-2^ s^-1^). Vx—violaxanthin, Ax—antheraxanthin, Zx—zeaxanthin, VAZ—the total pool of the xanthophyll cycle pigments. Data are presented as mean ± SE of values from three independent experiments (n = 3 for each experiment). Significant differences between mean values (ANOVA, Duncan’s test, *p* < 0.05) in each pigment analysis are indicated by different letters (a, b). The same or double letters (ab) indicate no significant differences between the means.; Table S3: Xanthophylls content (Vx, Ax, Zx) on dry weight (DW) and DEPS values in leaves of wild type (WT), AS-12, and XX-2 *Arabidopsis thaliana* plants grown at 90 μmol m^-2^ s^-1^ (0 h) and after 2, 4, 6, 8 h of moderately high light, MHL (at 400 μmol m^-2^ s^-1^) at dark-light-dark transitions. Dark1—after 1 h of darkness, light—after 1 h of light exposure (1000 µmol m^–2^ s^–1^), dark2—20 h-darkness following the light. Vx—violaxanthin, Ax—anteraxanthin, Zx—zeaxanthin. Data are presented as mean values ± SE (n = 3 for each experiment). Significant differences between mean values (Kruskal-Wallis’s test, *p* ˂ 0.05) are indicated by different letters (a, b, c). The same or double letters (ab, bc) indicate no significant differences between the means.; Table S4: Two-way ANOVA analysis for evaluation of the effect of MHL exposure time (time) and *AOX1a* expression level (Genotype) on DEPS change in *Arabidopsis thaliana* leaves.

## References

[1] Vanlerberghe G.C., Dahal K., Alber N.A., Chadee A. (2020). Photosynthesis, respiration and growth: A carbon and energy balancing act for alternative oxidase. Mitochondrion.

[2] Van Aken O. (2021). Mitochondrial redox systems as central hubs in plant metabolism and signalling. Plant Physiol..

[3] Noguchi K., Yoshida K. (2008). Interaction between photosynthesis and respiration in illuminated leaves. Mitochondrion.

[4] Dinakar C., Raghavendra A.S., Padmasree K. (2010). Importance of AOX pathway in optimizing photosynthesis under high light stress: Role of pyruvate and malate in activating AOX. Physiol. Plant..

[5] Zhang D.-W., Xu F., Zhang Z.-W., Chen Y.-E., Du J.-B., Jia S.-D., Yuan S., Lin H.-H. (2010). Effects of light on cyanide-resistant respiration and alternative oxidase function in Arabidopsis seedlings. Plant Cell Environ..

[6] Garmash E.V., Grabelnych O.I., Velegzhaninov I.O., Borovik O.A., Dalke I.V., Voinikov V.K., Golovko T.K. (2017). Light regulation of mitochondrial alternative oxidase pathway during greening of etiolated wheat seedlings. J. Plant Physiol..

[7] Giraud E., Ho L.H.M., Clifton R., Carroll A., Estavillo G., Tan Y.-F., Howell K.A., Ivanova A., Pogson B.J., Millar A.H. (2008). The absence of *ALTERNATIVE OXIDASE1a* in Arabidopsis results in acute sensitivity to combined light and drought stress. Plant Physiol..

[8] Watanabe C.K.A., Yamori W., Takahashi S., Terashima I., Noguchi K. (2016). Mitochondrial Alternative pathway-associated photoprotection of photosystem II is related to the photorespiratory pathway. Plant Cell Physiol..

[9] Yamada S., Ozaki H., Noguchi K. (2020). The Mitochondrial respiratory chain maintains the photosynthetic electron flow in *Arabidopsis thaliana* leaves under high-light stress. Plant Cell Physiol..

[10] Zhang D., Yuan S., Xu F., Zhu F., Yuan M., Ye H., Guo H., Lv X., Yin Y., Lin H. (2016). Light intensity affects chlorophyll synthesis during greening process by metabolite signal from mitochondrial alternative oxidase in *Arabidopsis*. Plant Cell Environ..

[11] Yoshida K., Terashima I., Noguchi K. (2011). How and why does the mitochondrial respiratory chain respond to light?. Plant Signal. Behav..

[12] Dahal K., Martyn G.D., Alber N.A., Vanlerberghe G.C. (2017). Coordinated regulation of photosynthetic and respiratory components is necessary to maintain chloroplast energy balance in varied growth conditions. J. Exp. Bot..

[13] Niyogi K.K., Grossman A.R., Björkman O. (1998). Arabidopsis mutants define a central role for the xanthophyll cycle in the regulation of photosynthetic energy conversion. Plant Cell..

[14] Jahns P., Latowski D., Strzalka K. (2009). Mechanism and regulation of the violaxanthin cycle: The role of antenna proteins and membrane lipids. Biochim. Biophys. Acta.

[15] Bartoli C.G., Yu J., Gómez F., Fernández L., McIntosh L., Foyer C.H. (2006). Inter-relationships between light and respiration in the control of ascorbic acid synthesis and accumulation in *Arabidopsis thaliana* leaves. J. Exp. Bot..

[16] Nunes-Nesi A., Sweetlove L.J., Fernie A.R. (2007). Operation and function of the tricarboxylic acid cycle in the illuminated leaf. Physiol. Plant..

[17] Zhang L.-T., Zhang Z.-S., Gao H.-Y., Meng X.-L., Yang C., Liu J.-G., Meng Q.-W. (2012). The mitochondrial alternative oxidase pathway protects the photosynthetic apparatus against photodamage in *Rumex K-1* leaves. BMC Plant Biol..

[18] Garmash E.V., Dymova O.V., Malyshev R.V., Plyusnina S.N., Golovko T.K. (2013). Developmental changes in energy dissipation in etiolated wheat seedlings during the greening process. Photosynthetica.

[19] Dahal K., Vanlerberghe G.C. (2018). Improved chloroplast energy balance during water deficit enhances plant growth: More crop per drop. J. Exp. Bot..

[20] Ho L.H.M., Giraud E., Uggalla V., Lister R., Clifton R., Glen A., Thirkettle-Watts D., Van Aken O., Whelan J. (2008). Identification of regulatory pathways controlling gene expression of stress-responsive mitochondrial proteins in Arabidopsis. Plant Physiol..

[21] Garmash E.V., Velegzhaninov I.O., Ermolina K.V., Rybak A.V., Malyshev R.V. (2020). Altered levels of *AOX1a* expression result in changes in metabolic pathways in *Arabidopsis thaliana* plants acclimated to low dose rates of ultraviolet B radiation. Plant Sci..

[22] Garmash E.V., Belykh E.S., Velegzhaninov I.O. (2021). The gene expression profiles of mitochondrial respiratory components in Arabidopsis plants with differing amounts of *ALTERNATIVE OXIDASE1a* under high intensity light. Plant Signal. Behav..

[23] Czechowski T., Stitt M., Altmann T., Udvardi M.K., Scheible W.-R. (2005). Genome-wide identification and testing of superior reference genes for transcript normalization in Arabidopsis. Plant Physiol..

[24] Umbach A.L., Fiorani F., Siedow J.N. (2005). Characterization of transformed Arabidopsis with altered alternative oxidase levels and analysis of effects on reactive oxygen species in tissue. Plant Physiol..

[25] Strodtkötter I., Padmasree K., Dinakar C., Speth B., Niazi P.S., Wojtera J., Voss I., Do P.T., Nunes-Nesi A., Fernie A.R. (2009). Induction of the AOX1D isoform of alternative oxidase in *A. thaliana* T-DNA insertion lines lacking isoform AOX1A is insufficient to optimize photosynthesis when treated with Antimycin A. Mol. Plant.

[26] Selinski J., Hartmann A., Deckers-Hebestreit G., Day D.A., Whelan J., Scheibe R. (2018). Alternative oxidase isoforms are differentially activated by tricarboxylic acid cycle intermediates. Plant Physiol..

[27] Florez-Sarasa I., Flexas J., Rasmusson A.G., Umbach A.L., Siedow J.N., Ribas-Carbo M. (2011). In vivo cytochrome and alternative pathway respiration in leaves of *Arabidopsis thaliana* plants with altered alternative oxidase under different light conditions. Plant Cell Environ..

[28] Kühn K., Yin G., Duncan O., Law S.R., Kubiszewski-Jakubiak S., Kaur P., Meyer E., Wang Y., Small C.C.D.F., Giraud E. (2015). Decreasing electron flux through the cytochrome and/or alternative respiratory pathways triggers common and distinct cellular responses dependent on growth conditions. Plant Physiol..

[29] Yoshida K., Shibata M., Terashima I., Noguchi K. (2010). Simultaneous determination of in vivo plastoquinone and ubiquinone redox states by HPLC-based analysis. Plant Cell Physiol..

[30] Morales L.M.M., Silva G.M.C., Santana D.B., Pireda S.F., Cogo A.J.D., Heringer A.S., Oliveira T.D.R., Reis R.S., dos Prado L.A.S., Oliveira A.V. (2022). Mitochondrial dysfunction associated with ascorbate synthesis in plants. Plant Physiol. Biochem..

[31] Wang D., Wang C., Li C., Song H., Qin J., Chang H., Fu W., Wang Y., Wang F., Li B. (2021). Functional relationship of Arabidopsis AOXs and PTOX revealed via transgenic analysis. Front. Plant. Sci..

[32] Müller-Moulé P., Havaux M., Niyogi K.K. (2003). Zeaxanthin Deficiency Enhances the high light sensitivity of an ascorbate-deficient mutant of Arabidopsis. Plant Physiol..

[33] Foyer C.H., Noctor G. (2011). Ascorbate and glutathione: The heart of the redox hub. Plant Physiol..

[34] Chew O., Whelan J., Millar A.H. (2003). Molecular definition of the ascorbate-glutathione cycle in Arabidopsis mitochondria reveals dual targeting of antioxidant defenses in plants. J. Biol. Chem..

[35] Carrie C., Giraud E., Whelan J. (2009). Protein Transport in Organelles: Dual targeting of proteins to mitochondria and chloroplasts. FEBS J..

[36] Jimenez A., Hernandez J.A., del Rio L.A., Sevilla F. (1997). Evidence for the presence of the ascorbate-glutathione cycle in mitochondria and peroxisomes of Pea leaves. Plant Physiol..

[37] Wang J., Vanlerberghe G.C. (2013). A lack of mitochondrial alternative oxidase compromises capacity to recover from severe drought stress. Physiol. Plant..

[38] Boyes D.C., Zayed A.M., Ascenzi R., McCaskill A.J., Hoffman N.E., Davis K.R., Gorlach J. (2001). Growth, stage-based phenotypic analysis of Arabidopsis: A model for high throughput functional genomics in plants. Plant Cell.

[39] Ye J., Coulouris G., Zaretskaya I., Cutcutache I., Rozen R., Madden T.L. (2012). Primer-BLAST: A tool to design target-specific primers for polymerase chain reaction. BMC Bioinf..

[40] Livak K.J., Schmittgen T.D. (2001). Analysis of relative gene expression data using real-time quantitative PCR and the 2^−ΔΔCT^ method. Methods.

[41] Bradford M.M. (1976). A rapid and sensitive method for the quantitation of microgram quantities of protein utilizing the principle of protein-dye binding. Anal. Biochem..

[42] Lichtenthaler H.K., Colowick S.P., Kaplan N.O. (1987). Chlorophylls and carotenoids: Pigments of photosynthetic biomembranes. Methods in Enzymology.

[43] Schindler C., Lichtenthaler H.K. (1996). Photosynthetic CO_2_-assimilation, chlorophyll fluorescence and zeaxanthin accumulation in field grown maple trees in the course of a sunny and a cloudy day. J. Plant Physiol..

[44] Kampfenkel K., Van Montagu M., Inzé D. (1995). Extraction and determination of ascorbate and dehydroascorbate from plant tissue. Anal. Biochem..

[45] Nakano Y., Asada K. (1981). Hydrogen Peroxide is scavenged by ascorbate-specific peroxidase in spinach chloroplasts. Plant Cell Physiol..

[46] Kley J., Heil M., Muck A., Svatos A., Boland W. (2010). Isolating intact chloroplasts from small Arabidopsis samples for proteomic studies. Anal. Biochem..

[47] Law M.Y., Charles S.A., Halliwell B. (1983). Glutathione and ascorbic acid in spinach (*Spinacia oleracea*) chloroplasts. The effect of hydrogen peroxide and of Paraquat. Biochem. J..

[48] Hossain M.A., Asada K. (1984). Inactivation of ascorbate peroxidase in spinach chloroplasts on dark addition of hydrogen peroxide: Its protection by ascorbate. Plant Cell Physiol..

