# Supplementary material for: AOX1a Expression in Arabidopsis thaliana Affects the State of Chloroplast Photoprotective Systems under Moderately High Light Conditions
Source: Plants (Basel). 2022 Nov 9;11(22):3030. doi: 10.3390/plants11223030 (PMC9697105; doi:10.3390/plants11223030)
Supplement: Supplementary file 1 [file plants-11-03030-s001.zip › Table S1.pdf]

Table S1. Specific leaf weight and chlorophylls (a+b) (Chl) content on dry weight (DW) and area in leaves of wild type (WT), AS-12, and XX-2 *Arabidopsis thaliana* plants grown at 90 μmol m^{-2} s^{-1} (0 h) and after 2-8 h of moderately high light, MHL (at 400 μmol m^{-2} s^{-1}).

Hours after MHL treatment, h	SLW, g DW dm^{-2}	Chl, mg g^{-1} DW	Chl, mg dm^{-2}
WT			
0	0.21±0.04	13.1±1.3	2.75±0.21 a
2	0.21±0.03	14.0±2.1	2.94±0.21 a
4	0.20±0.03	13.8±1.5	2.76±0.18 a
6	0.21±0.03	15.2±1.4	3.19±0.17 b
8	0.21±0.04	15.0±2.1	2.80±0.24 a
XX-2			
0	0.19±0.03	14.0±2.1	2.66±0.22 a
2	0.20±0.04	13.9±1.3	2.78±0.22 a
4	0.21±0.03	16.2±2.9	3.40±0.23 b
6	0.20±0.03	15.0±1.6	3.00±0.18 b
8	0.21±0.02	14.7±1.8	3.08±0.16 b
AS-12			
0	0.18±0.03	14.1±1.2	2.54±0.19 a
2	0.19±0.03	13.7±1.4	2.60±0.18 a
4	0.21±0.03	14.3±1.5	3.00±0.18 b
6	0.20±0.02	14.6±1.6	2.92±0.15 b
8	0.19±0.03	13.3±1.7	2.53±0.20 a

Data are presented as mean values ± SE of values from three independent experiments (n = 20 for SLW, n = 5 for Chl from each experiment). Significant differences between mean values (if there are) for each parameter are indicated by different letters (ANOVA, Dunkan's test, $P < 0.05$).
