# Supplementary material for: AOX1a Expression in Arabidopsis thaliana Affects the State of Chloroplast Photoprotective Systems under Moderately High Light Conditions
Source: Plants (Basel). 2022 Nov 9;11(22):3030. doi: 10.3390/plants11223030 (PMC9697105; doi:10.3390/plants11223030)
Supplement: Supplementary file 1 [file plants-11-03030-s001.zip › Table S2.pdf]

Table S2. Amounts of pigments (mg g^{-1}DW) of the xanthophylls cycle in leaves of wild type (WT), XX-2, and AS-12 *Arabidopsis thaliana* plants grown at 90 µmol m^{-2} s^{-1} (0 h) and after 2-8 h of moderately high light (at 400 µmol m^{-2} s^{-1}). Vx – violaxanthin, Ax – antheraxanthin, Zx – zeaxanthin, VAZ – the total pool of the xanthophyll cycle pigments.

Exposure to 400 µmol m^{-2} s^{-1}, h	Vx	Ax	Zx	VAZ
		WT		
0	0.142±0.015 b	0.018±0.002 a	0.022±0.011 a	0.182±0.042 ab
2	0.131±0.015 b	0.026±0.009 ab	0.034±0.007 ab	0.191±0.030 ab
4	0.141±0.010 b	0.028±0.008 b	0.036±0.009 ab	0.207±0.032 b
6	0.082±0.008 a	0.018±0.008 a	0.032±0.006 ab	0.132±0.024 a
8	0.084±0.007 a	0.030±0.002 b	0.032±0.008 ab	0.145±0.015 a
		XX-2		
0	0.132±0.016 b	0.015±0.001 a	0.020±0.015 a	0.167±0.052 a
2	0.112±0.005 b	0.025±0.003 ab	0.026±0.009 a	0.164±0.015 a
4	0.101±0.013 ab	0.029±0.009 b	0.029±0.011 a	0.158±0.029 a
6	0.087±0.004 a	0.016±0.004 a	0.029±0.008 a	0.133±0.016 a
8	0.071±0.007 a	0.028±0.003 b	0.043±0.006 b	0.149±0.017 a
		AS-12		
0	0.140±0.022 b	0.019±0.002 a	0.016±0.013 a	0.176±0.034 ab
2	0.092±0.027 ab	0.022±0.014 ab	0.026±0.005 a	0.141±0.043 a
4	0.130±0.009 b	0.046±0.013 b	0.053±0.016 b	0.229±0.032 b
6	0.081±0.013 a	0.032±0.003 b	0.050±0.019 b	0.157±0.031 a
8	0.089±0.016 a	0.031±0.007 b	0.027±0.007 a	0.146±0.008 a

Data are presented as mean ± SE of values from three independent experiments (n = 3 for each experiment). Significant differences between mean values (ANOVA, Duncan’s test, $P < 0.05$) in each pigment analysis are indicated by different letters (a, b). The same or double letters (ab) indicate no significant differences between the means.
