# Supplementary material for: AOX1a Expression in Arabidopsis thaliana Affects the State of Chloroplast Photoprotective Systems under Moderately High Light Conditions
Source: Plants (Basel). 2022 Nov 9;11(22):3030. doi: 10.3390/plants11223030 (PMC9697105; doi:10.3390/plants11223030)
Supplement: Supplementary file 1 [file plants-11-03030-s001.zip › Table S3.pdf]

Table S3. Xanthophylls content (Vx, Ax, Zx) on dry weight (DW) and DEPS values in leaves of wild type (WT), AS-12, and XX-2 *Arabidopsis thaliana* plants grown at 90 μmol m^{-2} s^{-1} (0 h) and after 2, 4, 6, 8 h of moderately high light, MHL (at 400 μmol m^{-2} s^{-1}) at dark-light-dark transitions. Dark1 - after 1 h of darkness, light - after 1 h of light exposure (1000 μmol m^{-2} s^{-1}), dark2 – 20 h-darkness following the light. Vx – violaxanthin, Ax – anteraxanthin, Zx - zeaxanthin.

Hours after MHL treatment, h	Light intensity	Vx, mg g^{-1}DW	Ax, mg g^{-1}DW	Zx, mg g^{-1}DW	Vx+ Ax+ Zx, mg g^{-1}DW	DEPS, %
WT						
0	dark1	0.054±0.021 ^{a}	0.011±0.002 ^{ab}	0.015±0.001 ^{b}	0.080±0.024 ^{a}	27±8 ^{a}
	light	0.048±0.003 ^{a}	0.021±0.006 ^{c}	0.003±0.001 ^{a}	0.072±0.009 ^{a}	19±1 ^{ab}
	dark2	0.055±0.003 ^{a}	0.006±0.001 ^{a}	0.016±0.002 ^{b}	0.077±0.005 ^{a}	24±1 ^{a}
2	dark1	0.071±0.003 ^{a}	0.009±0.001 ^{a}	0.005±0.001 ^{a}	0.084±0.005 ^{a}	11±1 ^{a}
	light	0.072±0.003 ^{a}	0.012±0.001 ^{b}	0.021±0.011 ^{b}	0.105±0.012 ^{b}	25±8 ^{b}
	dark2	0.070±0.001 ^{a}	0.008±0.001 ^{a}	0.019±0.003 ^{b}	0.096±0.003 ^{ab}	23±3 ^{b}
4	dark1	0.055±0.005 ^{ab}	0.009±0.002 ^{ab}	0.020±0.003 ^{a}	0.084±0.010 ^{ab}	29±1 ^{a}
	light	0.047±0.011 ^{a}	0.011±0.003 ^{bc}	0.015±0.003 ^{a}	0.073±0.012 ^{a}	28±5 ^{a}
	dark2	0.072±0.021 ^{b}	0.007±0.001 ^{a}	0.015±0.006 ^{a}	0.094±0.019 ^{b}	21±9 ^{a}
6	dark1	0.074±0.003 ^{b}	0.012±0.003 ^{a}	0.015±0.006 ^{a}	0.101±0.003 ^{b}	21±4 ^{a}
	light	0.047±0.015 ^{a}	0.005±0.002 ^{a}	0.017±0.007 ^{b}	0.069±0.023 ^{a}	28±3 ^{b}
	dark2	0.042±0.011 ^{a}	0.010±0.002 ^{b}	0.005±0.003 ^{a}	0.057±0.015 ^{a}	18±2 ^{a}
8	dark1	0.062±0.002 ^{b}	0.009±0.001 ^{a}	0.017±0.002 ^{a}	0.088±0.002 ^{a}	25±2 ^{a}
	light	0.040±0.004 ^{a}	0.020±0.003 ^{b}	0.021±0.005 ^{a}	0.080±0.008 ^{a}	37±9 ^{a}
	dark2	0.059±0.004 ^{b}	0.009±0.001 ^{a}	0.016±0.001 ^{a}	0.084±0.006 ^{a}	25±1 ^{a}
XX-2						
0	dark1	0.046±0.014 ^{a}	0.004±0.001 ^{a}	0.012±0.005 ^{a}	0.062±0.019 ^{a}	22±2 ^{a}
	light	0.037±0.004 ^{a}	0.010±0.003 ^{b}	0.011±0.004 ^{a}	0.057±0.008 ^{a}	27±4 ^{a}
	dark2	0.041±0.002 ^{a}	0.005±0.002 ^{a}	0.011±.006 ^{a}	0.058±0.004 ^{a}	24±7 ^{a}
2	dark1	0.057±0.016 ^{a}	0.009±0.004 ^{ab}	0.012±0.005 ^{a}	0.078±0.025 ^{a}	32±4 ^{a}
	light	0.063±0.001 ^{a}	0.017±0.001 ^{b}	0.025±0.001 ^{b}	0.105±0.001 ^{a}	44±1 ^{b}
	dark2	0.069±0.001 ^{a}	0.006±0.001 ^{a}	0.010±0.002 ^{a}	0.085±0.003 ^{a}	22±1 ^{a}
4	dark1	0.104±0.008 ^{b}	0.011±0.002 ^{a}	0.015 ±0.002 ^{a}	0.130±0.012 ^{b}	15±1 ^{a}
	light	0.057±0.003 ^{a}	0.025±0.001 ^{c}	0.029±0.002 ^{c}	0.111±0.005 ^{ab}	38±1 ^{c}
	dark2	0.091±0.023 ^{b}	0.007±0.003 ^{a}	0.011±0.002 ^{a}	0.110±0.027 ^{ab}	14±2 ^{a}
6	dark1	0.042±0.011 ^{ab}	0.008±0.002 ^{a}	0.019±0.007 ^{b}	0.068±0.020 ^{a}	21±3 ^{b}
	light	0.032±0.008 ^{a}	0.011±0.002 ^{a}	0.025±0.006 ^{c}	0.067±0.017 ^{a}	32±1 ^{c}
	dark2	0.053±0.001 ^{b}	0.007±0.001 ^{a}	0.012±0.001 ^{ab}	0.072±0.002 ^{a}	15±2 ^{a}
8	dark1	0.062±0.010 ^{a}	0.010±0.002 ^{a}	0.012±0.001 ^{a}	0.083±0.012 ^{a}	20±1 ^{b}
	light	0.039±0.007 ^{c}	0.023±0.002 ^{c}	0.022±0.001 ^{c}	0.084±0.009 ^{a}	40±3 ^{d}
	dark2	0.017±0.002 ^{b}	0.001±0.000 ^{b}	0.001±0.000 ^{a}	0.019±0.002 ^{b}	8±2 ^{a}
AS-12						
0	dark1	0.098±0.004 ^{a}	0.009±0.001 ^{a}	0.040±0.008 ^{b}	0.147±0.013 ^{a}	30±3 ^{a}
	light	0.082±0.029 ^{a}	0.023±0.006 ^{a}	0.034±0.014 ^{ab}	0.139±0.048 ^{a}	32±2 ^{a}
	dark2	0.089±0.031 ^{a}	0.010±0.003 ^{a}	0.021±0.010 ^{a}	0.120±0.044 ^{a}	21±3 ^{c}
2	dark1	0.137±0.012 ^{a}	0.013±0.001 ^{a}	0.022±0.004 ^{a}	0.172±0.010 ^{a}	25±8 ^{a}
	light	0.102±0.042 ^{a}	0.030±0.013 ^{b}	0.024±0.017 ^{a}	0.156±0.072 ^{a}	44±5 ^{b}
	dark2	0.135±0.002 ^{a}	0.014±0.001 ^{a}	0.007±0.002 ^{ab}	0.156±0.003 ^{a}	22±1 ^{a}
4	dark1	0.141±0.025 ^{a}	0.015±0.003 ^{a}	0.023±0.008 ^{a}	0.179±0.034 ^{a}	19±3 ^{a}
	light	0.134±0.008 ^{a}	0.029±0.004 ^{b}	0.021±0.002 ^{a}	0.184±0.004 ^{a}	41±4 ^{b}

	dark2	0.118±0.015 [a]	0.013±0.002 [a]	0.022±0.013 [a]	0.153±0.029 [a]	14±2 [a]
6	dark1	0.149±0.011 [c]	0.020±0.006 [a]	0.028±0.006 [a]	0.197±0.008 [a]	17±3 [bc]
	light	0.097±0.007 [b]	0.038±0.009 [a]	0.062±0.014 [a]	0.197±0.029 [a]	23±6 [c]
	dark2	0.126±0.002 [a]	0.018±0.001 [a]	0.014±0.003 [a]	0.158±0.002 [a]	9±1 [a]
8	dark1	0.093±0.022 [a]	0.019±0.006 [ab]	0.026±0.016 [a]	0.139±0.034 [a]	17±2 [a]
	light	0.065±0.015 [a]	0.027±0.007 [b]	0.050±0.020 [a]	0.141±0.040 [a]	19±2 [a]
	dark2	0.098±0.002 [a]	0.014±0.001 [a]	0.023±0.003 [a]	0.135±0.007 [a]	18±6 [a]

Data are presented as mean values ± SE (n = 3 for each experiment). Significant differences between mean values (Kruskal-Wallis's test, $p < 0.05$) are indicated by different letters (a, b, c). The same or double letters (ab, bc) indicate no significant differences between the means.
