# Supplementary material for: AOX1a Expression in Arabidopsis thaliana Affects the State of Chloroplast Photoprotective Systems under Moderately High Light Conditions
Source: Plants (Basel). 2022 Nov 9;11(22):3030. doi: 10.3390/plants11223030 (PMC9697105; doi:10.3390/plants11223030)
Supplement: Supplementary file 1 [file plants-11-03030-s001.zip › Table S4.pdf]

Table S4. Two-way ANOVA analysis for evaluation of the effect of MHL exposure time (time) and *AOX1a* expression level (Genotype) on DEPS change in *Arabidopsis thaliana* leaves.

	SS	Degree of fr.	MS	F	p
Intersept	1281,896	1	1281,896	2197,537	0,000000
Genotype	7.215	2	3.607	6.184	0.002779
time	93.141	4	23.285	39.917	0.000000
Genotype*time	87.748	8	10.969	18.803	0.000000
Error	70.000	120	0.583		
