# Supplementary figures and images for: AOX1a Expression in Arabidopsis thaliana Affects the State of Chloroplast Photoprotective Systems under Moderately High Light Conditions
Source: Plants (Basel). 2022 Nov 9;11(22):3030. doi: 10.3390/plants11223030 (PMC9697105; doi:10.3390/plants11223030)

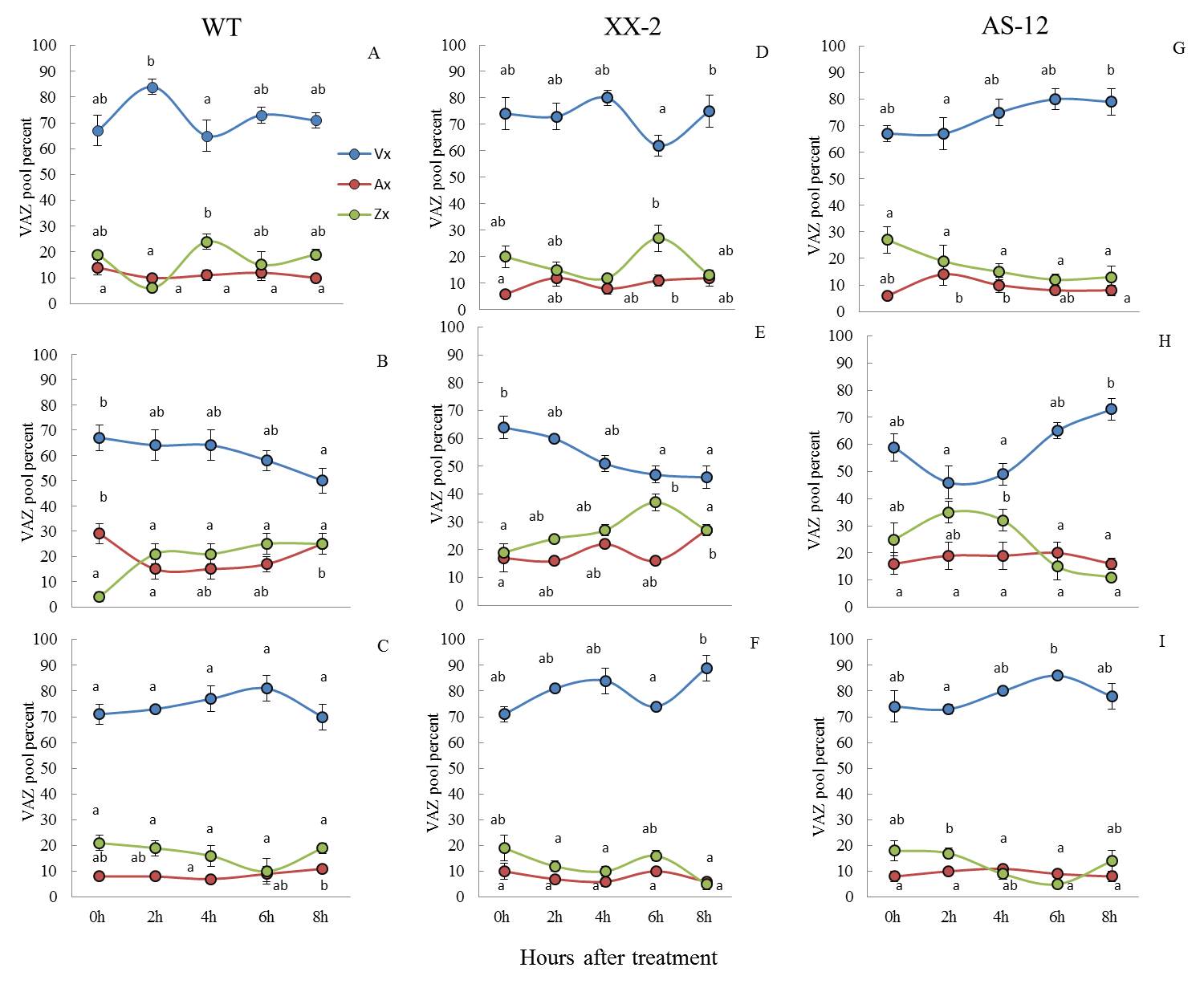

Supplement: Supplementary file 1 [file plants-11-03030-s001.zip › Figure S1.jpg]

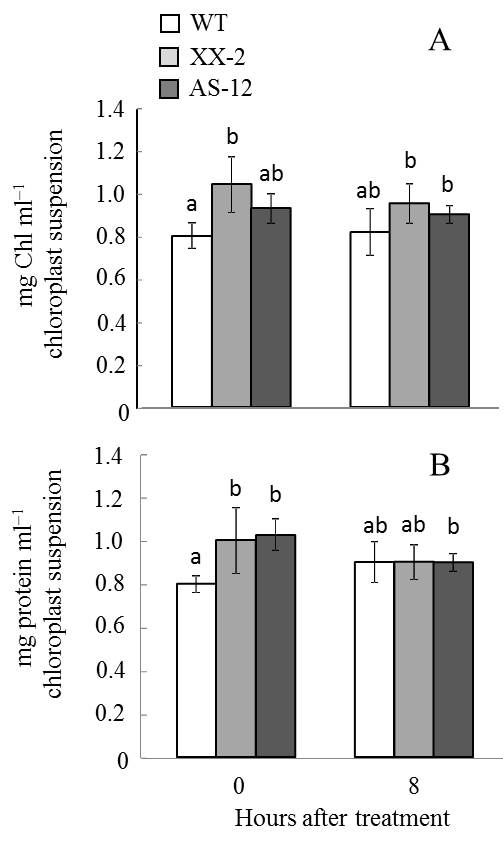

Supplement: Supplementary file 1 [file plants-11-03030-s001.zip › Figure S2.jpg]

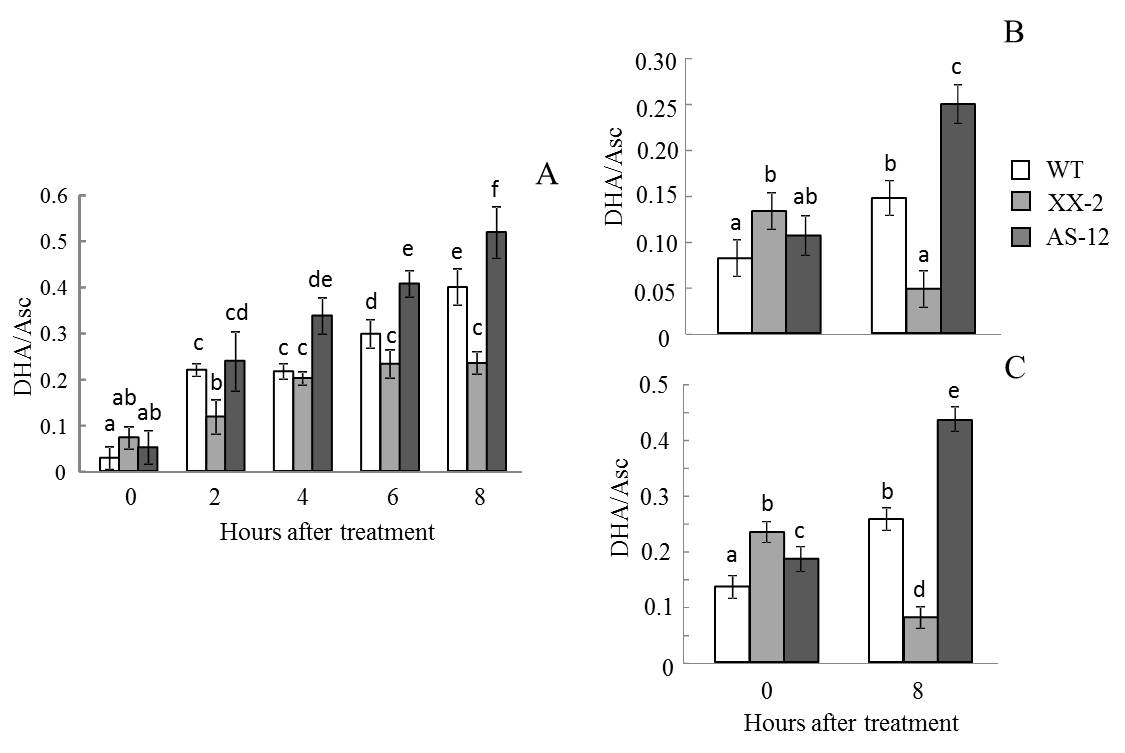

Supplement: Supplementary file 1 [file plants-11-03030-s001.zip › Figure S3.jpg]
